# Causal relationship between diabetes mellitus, glycemic traits and Parkinson’s disease: a multivariable mendelian randomization analysis

**DOI:** 10.1186/s13098-024-01299-8

**Published:** 2024-03-05

**Authors:** Qitong Wang, Benchi Cai, Lifan Zhong, Jitrawadee Intirach, Tao Chen

**Affiliations:** 1 Department of Neurology, Hainan General Hospital, Hainan Afliated Hospital of Hainan Medical University, 570311 Haikou, Hainan China; 2 Hainan Provincial Bureau of Disease Prevention and Control, 570100 Haikou, China

**Keywords:** Diabetes mellitus, Parkinson's disease, Mendelian randomization, Genetic epidemiology

## Abstract

**Background:**

Observational studies have indicated an association between diabetes mellitus (DM), glycemic traits, and the occurrence of Parkinson’s disease (PD). However, the complex interactions between these factors and the presence of a causal relationship remain unclear. Therefore, we aim to systematically assess the causal relationship between diabetes, glycemic traits, and PD onset, risk, and progression.

**Method:**

We used two-sample Mendelian randomization (MR) to investigate potential associations between diabetes, glycemic traits, and PD. We used summary statistics from genome-wide association studies (GWAS). In addition, we employed multivariable Mendelian randomization to evaluate the mediating effects of anti-diabetic medications on the relationship between diabetes, glycemic traits, and PD. To ensure the robustness of our findings, we performed a series of sensitivity analyses.

**Results:**

In our univariable Mendelian randomization (MR) analysis, we found evidence of a causal relationship between genetic susceptibility to type 1 diabetes (T1DM) and a reduced risk of PD (OR = 0.9708; 95% CI: 0.9466, 0.9956; P = 0.0214). In our multivariable MR analysis, after considering the conditions of anti-diabetic drug use, this correlation disappeared with adjustment for potential mediators, including anti-diabetic medications, insulin use, and metformin use.

**Conclusion:**

Our MR study confirms a potential protective causal relationship between genetically predicted type 1 diabetes and reduced risk of PD, which may be mediated by factors related to anti-diabetic medications.

**Supplementary Information:**

The online version contains supplementary material available at 10.1186/s13098-024-01299-8.

## Introduction

Diabetes mellitus (DM) and Parkinson’s disease (PD) are disorders associated with aging, and their prevalence is increasing worldwide. In the past few decades, the global number of adult diabetes patients has increased from 108 million in 1980 to 422 million in 2014 [1]. At the same time, the age-standardized prevalence of diabetes in men increased from 4.3 to 9.0% and in women from 5.0–7.9% [1]. It is estimated that by 2045, the number of diabetes patients will increase to 783 million [2]. Parkinson’s disease (PD) is also a rapidly developing neurodegenerative disease, with a global average prevalence of 1–2‰ [3]. With the exacerbation of population aging, the burden of PD will become even heavier [4]. According to statistics, from 1990 to 2016, the incidence, disability burden, and mortality related to Parkinson’s disease have more than doubled. Furthermore, a global survey of neurological diseases shows that PD may be the fastest-growing neurological disease globally [5]. In recent years, the role of DM in neurodegeneration has grown special interest not only as a contributing factor to disease onset but also as a modifying factor of motor and nonmotor symptoms [6].

Some epidemiological studies suggest an association between diabetes and PD, but the results are not entirely consistent with some positive correlation studies [7]. A recent meta-analysis included 15 cohort studies (including over 86,000 PD cases and nearly 30 million participants), reporting a 27% increased risk of PD in patients with diabetes [8]. An earlier meta-analysis included 7 cohort studies (including 1,761,632 patients) and found that the risk of PD in patients with diabetes also increased by 38% [9]. It is worth noting that the results of a few case-control studies suggest that diabetes may reduce the risk of PD [10, 11]. This difference may be attributed to heterogeneity, confounding factors, and biases between studies (such as inclusion and recall biases) [10]. Therefore, the causal relationship between diabetes and PD is still controversial.

Factors such as ethical and moral constraints, methodological confounding, and reverse causality contribute to the lack of high-quality randomized controlled trial (RCT) data in observational studies. However, Mendelian randomization (MR) provides a promising alternative. MR, which conceptually resembles a randomized controlled trial, is based on the principle of random allocation of genetic variations during meiosis. This random allocation makes genetic variations independent of many factors influencing observational studies. To investigate the causal relationship between genetic liability to diabetes and glycemic traits with Age at onset (AAO), risk of PD, and progression (UPDRS3/MMSE/MOCA), we conducted univariable Mendelian randomization (UVMR). UVMR allows us to examine the potential causal effects of genetic variations on these outcomes. Considering the everyday use of clinical anti-diabetic medications in diabetes management, we implemented multivariable Mendelian randomization (MVMR) to account for biases induced by the concomitant use of anti-diabetic drugs. This approach allows us to control for the potential confounding effects of these medications on the observed associations.

## Materials and methods

### Study design

We used the two-sample MR method to investigate the potential causal relationship between diabetes, blood glucose traits, and PD. Specifically, we retrieved summary genetic data for exposure and outcome from two independent samples based on strict genetic instrumental variables (IVs) criteria, avoiding bias caused by overlap [12]. Finally, we used rigorously selected SNPs for our final MR analysis. Currently, the GWAS database of the European population is the largest publicly available, so we focused on studying participants of European ancestry.

**Fig. 1:**
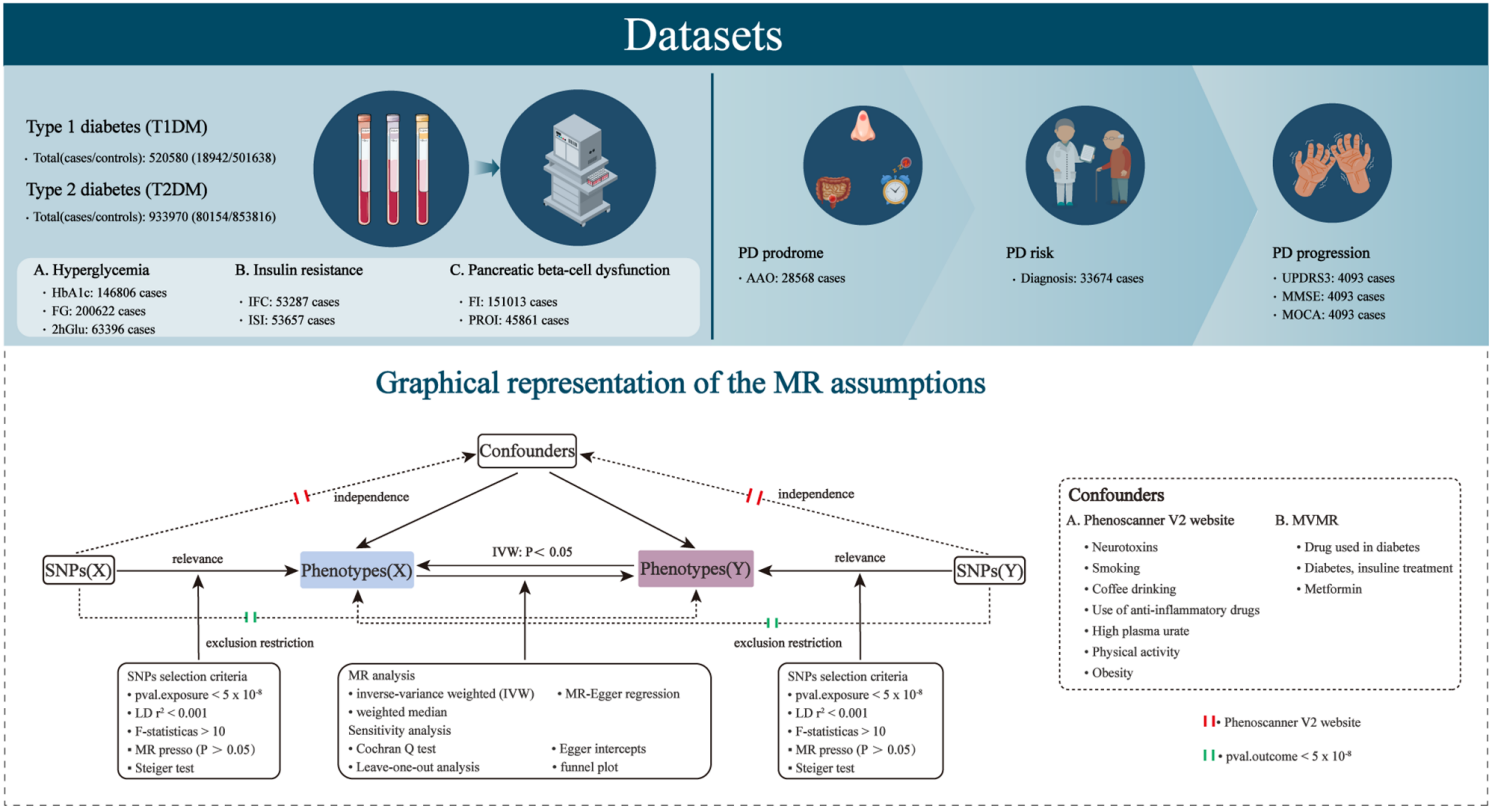
Graphical representation of MR assumptions

All MR analyses in our study need to meet three fundamental assumptions: (I) Instrumental variables are closely related to the exposure; (II) Instrumental variables are independent of confounding factors; (III) Instrumental variables only affect the outcome through the exposure (see Fig. 1) [13]. The analysis was conducted using the TwoSampleMR package (version 0.5.6) in R software (version 4.2.2).

### Data source

All data for this study were based on publicly available GWAS summary results (see Table 1). The T1DM data were obtained from a large GWAS summary dataset with a sample size of 520,580 (18,942 cases and 501,638 controls) [14]. The T2DM data were obtained from the Diabetes Genetics Replication and Meta-analysis (DIAGRAM) consortium, one of the most extensive collaborative efforts focused on characterizing the genetic basis of T2DM. This GWAS study involved 933,970 individuals of European ancestry, including 80,154 T2DM cases and 853,816 controls [15]. Additionally, data for other relevant traits such as glycated hemoglobin levels [14] (N~146,806), fasting glucose [14] (N~200,622), two-hour glucose [14](N~63,396), insulin fold change during an oral glucose tolerance test (adjusted for BMI) [16] (N~53,287), modified Stumvoll insulin sensitivity index (adjusted for BMI) [16] (N~53,657), fasting insulin [17] (N~151,013), and proinsulin [18] (N~45,861) were obtained from The Meta-Analyses of Glucose and Insulin-related traits Consortium (MAGIC).

**Table 1:** Details of the data sources used in this study

Phenotype	Abre.	Traits	Source	Sample size Total (cases/ controls)	Ancestry	Reference
**Diabetes Phenotypes & Glycemic Traits**						
Type 1 diabetes	T1DM	Type 1 diabetes	NA	520,580 (18,942/501,638)	European	Chiou et al. [14]
Type 2 diabetes	T2DM	Type 2 diabetes	NA	933,970 (80,154/853,816)	European	Mahajan et al. [15]
Glycated hemoglobin levels	HbA1c	Glucose tolerance test	MAGIC	146,806	European	Chen et al. [17]
Fasting glucose	FG	Glucose tolerance test	MAGIC	200,622	European	Chen et al. [17]
Two-hour glucose	2hGlu	Glucose tolerance test	MAGIC	63,396	European	Chen et al. [17]
Insulin fold change during an oral glucose tolerance test (adjusted for BMI)	IFC	Insulin resistance	MAGIC	53,287	European	Williamson et al. [16]
Modified Stumvoll Insulin Sensitivity Index (adjusted for BMI)	ISI	Insulin resistance	MAGIC	53,657	European	Williamson et al. [16]
Fasting insulin	FI	Pancreatic β-cell dysfunction	MAGIC	151,013	European	Chen et al. [17]
Proinsulin	PROI	Pancreatic β-cell dysfunction	MAGIC	45,861	European	Broadaway et al. [18]
**Parkinson’s disease phenotypes**						
Parkinson’s disease risk	PD risk	PD risk	IPDGC	482,730 (33,674/449,056)	European	Nalls et al. [20]
Age at onset of Parkinson’s disease	AAO	PD prodrome	IPDGC	28,568	European	Blauwendraat et al. [19]
UPDRS3	NA	PD progression	IPDGC	4093	European	Iwaki et al. [21]
MMSE	NA	PD progression	IPDGC	4093	European	Iwaki et al. [21]
MOCA	NA	PD progression	IPDGC	4093	European	Iwaki et al. [21]
**Anti-diabetic drugs phenotypes**						
Drugs used in diabetes	NA	Antidiabetic drugs	UK Biobank	305,913 (15,272/290,641)	European	Wu et al. [23]
Diabetes, insulin treatment	NA	Anti-diabetic drugs	FinnGen	218,792 (29,071/189,721)	European	Kurki et al. [24]
Metformin	NA	Anti-diabetic drugs	FinnGen	462,933 (11,552/451,381)	European	Kurki et al. [24]

MAGIC: Meta-Analyses of Glucose and Insulin-related Traits Consortium; IPDGC: International Parkinson’s Disease Genomics Consortium; UPDRS3, Unified Parkinson’s Disease Rating Scale part III; MMSE, Mini-Mental State Examination; MoCA, Montreal Cognitive Assessment

PD-related phenotypic data AAO [19] (N~28,568), PD risk [20] (N~482,730), UPDRS3/MMSE/ MOCA [21] (N~4093) were obtained from the International Parkinson’s Disease Genomics Consortium (IPDGC) [22].

The phenotype data related to anti-diabetic drugs were obtained from the IEU Open GWAS project (https://gwas.mrcieu.ac.uk/), including Drugs used in diabetes [23](N~305,913), Diabetes, insulin treatment [24] (N~218,792), Metformin [24] (N~462,933).

All studies have obtained ethical approval from their respective institutional review boards and include written informed consent from the participants and strict quality control. Since all analyses in this paper are based on publicly available summary data, ethical approval from institutional review boards is not required for this study.

### Selection of genetic instruments and data harmonization

Select genetic instruments based on the following criteria (see Table S1): I. Choose genetic variants that are closely associated with the exposure (P < 5 × 10^− 8^, F-statistic > 10) and are independent [linkage disequilibrium (LD) r^2^ < 0.001, Window size = 1 Mb]. II. Remove SNPs closely associated with the outcome (p < 5 × 10^− 8^). III;. Apply the MR Pleiotropy RESidual Sum and Outlier (MR-PRESSO) test to remove potential outliers before each MR analysis (P < 0.05). III;. To determine whether SNPs are associated with potential risk factors, we searched all SNPs in PhenoScanner (Version 2, http://www.phenoscanner.medschl.cam.ac.uk/) [25, 26]. We removed SNPs associated with the disease or potential risk factors related to PD, including neurotoxins, smoking, coffee drinking, use of anti-inflammatory drugs, high plasma urate, physical activity, and obesity (see Table S2) [27]. The remaining SNPs were used in the MR analysis.

### MR analysis

To avoid potential pleiotropic effects, we employed three different MR methods (inverse-variance weighted (IVW), MR-Egger regression, weighted median, and weighted mode) to assess the bidirectional causal effects between diabetes and PD. The results from the IVW method were used as the primary outcome. MR-Egger and weighted median complemented the IVW estimates (P < 0.05 indicating a causal relationship between exposure and outcome). IVW is a commonly used primary method in MR studies, which combines all Wald ratios of each SNP to obtain an overall estimate [28]. IVW assumes that all genetic variations are valid, making it the most efficient MR estimation method, but it is also prone to pleiotropic bias. Conversely, MR-Egger believes the instrument strength is independent of the direct effect (internal) and negligible measurement error (NOME) [29]. Weighted median assumes that at least half of the instruments are valid [30].

To demonstrate the reliability of our results, we conducted a series of sensitivity analyses to assess potential confounding factors. These factors include horizontal pleiotropy, heterogeneity, and reverse causality in the study. We utilized Cochran’s Q test and a funnel plot to measure potential heterogeneity. Cochran’s Q statistic evaluates heterogeneity among genetic variations, with a significance level of P < 0.05, indicating the presence of heterogeneity. To estimate horizontal pleiotropy, we performed the MR-Egger Intercept test. A significance level of P < 0.05 indicates the presence of horizontal pleiotropy [31]. We employed Steiger’s directional test to detect variations that are more strongly associated with the outcome than the exposure [32]. If the Steiger test provides evidence of a stronger association for specific genetic instruments, we repeated the analysis after excluding these variations [33]. To assess potential directional pleiotropy, we utilized a funnel plot. Additionally, we conducted a leave-one-out study to evaluate whether the causal relationship depends on or is biased by any individual SNP. Furthermore, we performed reverse MR analysis on results with P_IVW_ < 0.05 to assess whether or not the results are influenced by reverse causality.

To address potential confounding caused by the combined use of diabetes, blood glucose traits, and anti-diabetic medication in assessing Parkinson’s disease-related phenotypes, we employed the Multivariable MR (MVMR) method [34]. Overall, these sensitivity analyses enhance the reliability of our findings by accounting for potential confounding factors and providing a more comprehensive assessment of the relationship between the variables of interest.

## Results

**Fig. 2:**
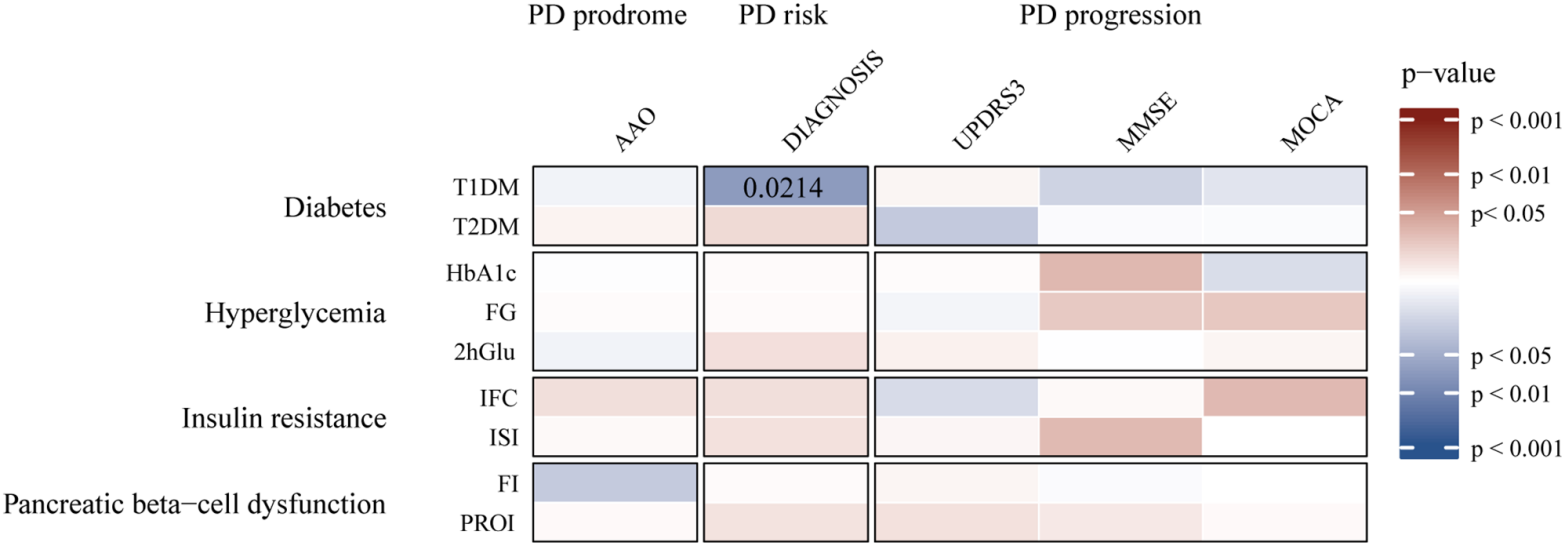
IVW estimates from Diabetes Mellitus, Glycemic traits on PD phenotypes. The color of each block represents the IVW-derived P-values for each MR analysis, examining the association between Diabetes mellitus, Glycemic traits, and PD (red indicates a positive association, and blue indicates a negative association). PD refers to Parkinson’s disease, AAO stands for Age at onset, UPDRS3 stands for Unified Parkinson’s Disease Rating Scale part III, MMSE stands for Mini-Mental State Examination, and MoCA stands for Montreal Cognitive Assessment

**Fig. 3:**
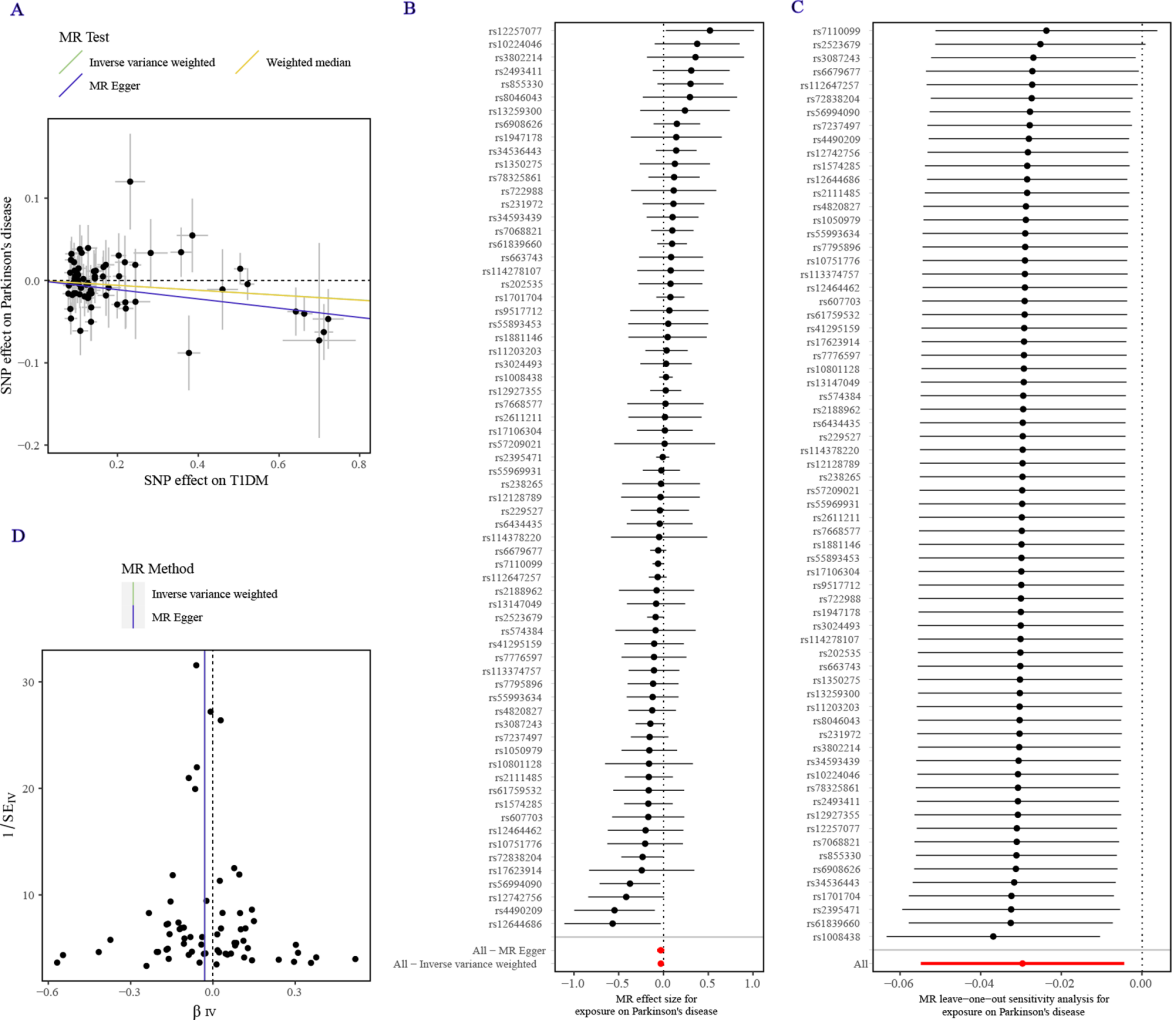
Mendelian randomization (MR) analysis results for T1DM and its impact on Parkinson’s disease (PD) risk. (**A**) A scatter plot illustrating the potential effects of single nucleotide polymorphisms (SNPs) on T1DM and PD risk using IVW, MR-Egger, and weighted median methods. The slope of the fitted lines represents the estimated MR effect per method, while the 95% CI for the effect size on T1DM is shown as vertical lines, and the 95% CI for PD risk is shown as horizontal lines. (**B**) A funnel plot for T1DM shows the estimation using the inverse of the standard error of the causal estimate with each SNP as a tool. The vertical line depicts the estimated causal effect obtained using IVW and MR-Egger methods. (**C**) A forest plot demonstrating the impact of each SNP in the MR analysis. (**D**) A forest plot presenting the results of the leave-one-out sensitivity analysis, where each SNP in the instrument was iteratively removed to check the stability of the result

Univariate conventional MR analysis showed a correlation between the genetic prediction of T1DM and a reduced risk of PD (IVW OR = 0.9708; 95% CI: 0.9466, 0.9956; P = 0.0214) (see Figs. 2 and 3; Table 2, S3). The estimated associations from MR Egger and Weighted median analyses were consistent with the observed associations in the primary study, but the confidence intervals were often imprecise. It is worth noting that these sensitivity methods have lower statistical power than IVW because they rely on more stringent assumptions; thus, their results are expected to provide weaker statistical evidence but cannot offer effect sizes. There is no statistical evidence for an impact of T2DM on the risk of PD (IVW OR = 1.0292; 95% CI: 0.9714, 1.0905; P = 0.3284). Furthermore, there is no statistical evidence to suggest an association between diabetes, glycemic traits, and other phenotypes of PD.

**Table 2:** Main results of the MR analysis and sensitivity analysis

**Outcome**	**N**		MR analysis		Heterogeneity		MR-Egger pleiotropy		MR PRESSO	Directionality	
	SNVs				Test		Test		Test	Test	
		Method	Estimate (95% CI)	*P*	Q value	*P*	Egger intercept	*P*	Global Test P	Correct directionaliy	*P*
**Type 1 diabetes (T1DM)**											
PD risk^*^	68	IVW	0.9708(0.9466, 0.9956)	0.0214	69.3901	0.3968	0.0002	0.9704	0.3885	TRUE	0
PD AAO	49	IVW	-0.0432(-0.2235, 0.1369)	0.6379	36.5035	0.8875	-0.0132	0.6908	0.8941	TRUE	0
UPDRS3	29	IVW	0.0081(-0.0347, 0.0508)	0.712	6.2855	1	0.0059	0.5376	1	TRUE	0.6823
MMSE	31	IVW	-0.0502(-0.1265, 0.0261)	0.1969	8.1621	1	0.0018	0.9106	1	TRUE	0.0654
MOCA	23	IVW	-0.0983(-0.3123, 0.1157)	0.3678	7.9993	0.9972	0.0037	0.9445	0.9951	TRUE	0.8692
**Type 2 diabetes (T2DM)**											
PD risk	154	IVW	1.0292(0.9714, 1.0905)	0.3284	194.3979	0.0132	-0.0077	0.0921	0.0262	TRUE	0
PD AAO	119	IVW	0.0780(-0.2895, 0.4455)	0.6774	67.9223	0.9999	0.03	0.3063	1	TRUE	0
UPDRS3	49	IVW	-0.0796(-0.1834, 0.0243)	0.1331	7.9237	1	0.01	0.2797	1	FALSE	0.0017
MMSE	46	IVW	-0.0194(-0.2116, 0.1728)	0.8431	4.1319	1	0.004	0.8081	1	FALSE	0
MOCA	45	IVW	-0.0532(-0.6078, 0.5014)	0.8508	5.8716	1	0.0415	0.3616	1	FALSE	0.0003

N SNPs: number of single nucleotide polymorphisms in the instrument. IVW: Inverse variance weighted. MR: Mendelian randomization. MR-PRESSO: Mendelian Randomization Pleiotropy RESidual Sum and Outlier. OR: Odds ratio. CI: confidence interval. Beta: MR effect estimate. Se: standard error of MR effect estimate. P: P-value. PD: Parkinson’s Disease; AAO: Age at onset of Parkinson’s disease; UPDRS3: Unified Parkinson’s Disease Rating Scale part III. MMSE: Mini-Mental State Examination. MoCA: Montreal Cognitive Assessment. Describing PD risk results using OR (95% CI) and UPDRS3/MMSE/MOCA results using Bete ± se

We conducted a series of sensitivity tests to assess the accuracy of the optimistic estimates. These tests included Cochran’s Q-test, MR-Egger intercept, leave-one-out analysis, and funnel plot. The results of Cochran’s Q-test indicated no heterogeneity (P = 0.3968), suggesting that the studies included in our calculation were consistent. Additionally, the MR-Egger intercept test (P = 0.9704) did not detect potential horizontal pleiotropy, further supporting the reliability of our findings. Furthermore, the leave-one-out analysis results indicated that the causal effect was not driven by a single instrumental variable, suggesting that the observed association was robust. The symmetrical funnel plot also stated the results’ reliability, suggesting minimal publication bias. We conducted directionality checks using Steiger’s analysis to validate our findings further. These checks did not indicate a violation of the observed causal relationship, strengthening the evidence for our significant associations. Moreover, we performed reverse MR analysis to assess the influence of reverse causality on our results. The analysis showed that the results were unlikely to be influenced by reverse causality (IVW OR = 0.9347; 95% CI: 0.8657, 1.0092; P = 0.0844), providing additional support for the robustness of our findings(see Table S4).

In the context of MVMR, we evaluated the genetic risk of T1DM in combination with anti-diabetic drugs (see Table 3, 5). After adjusting for phenotypes related to anti-diabetic medications, such as drugs used in diabetes (IVW OR = 0.9812; 95% CI: 0.9324, 1.0325; P = 0.4740), diabetes, insulin treatment (IVW OR = 0.9822; 95% CI: 0.9463, 1.0194; P = 0.3380), and Metformin (IVW OR = 1.0000; 95% CI: 0.9825, 1.0178; P = 0.9930), the correlation between T1DM and PD risk was no longer significant. This suggests that the observed association between T1DM and PD risk may be confounded by the use of anti-diabetic drugs. The estimated associations from MR Egger and Weighted median analyses consistently aligned with the associations observed in IVW. Moreover, Cochran’s Q-test and MR-Egger intercept test did not reveal potential heterogeneity and pleiotropy, further supporting the robustness of our findings.

**Table 3:** Multivariable MR results after adjusting for the anti-diabetic drug

**Exposure**	Outcome	Adjustments	N SNP	Methods	Causal effect		Heterogeneity		Pleiotropy	
					OR (95%CI)	p	Q value	p	Intercept	p
TIDM	PD risk	Drug used in diabetes	134	IVW	0.9812(0.9324, 1.0325)	0.474	61.8836	0.2742	61.5	0.2547
		Diabetes, insulin treatment	126	IVW	0.9822(0.9463, 1.0194)	0.338	63.978	0.89	63.92387	0.8747
		Metformin	126	IVW	1.0000(0.9825, 1.0178)	0.993	117.526	0.1395	117.5186	0.1249

N SNPs: number of single nucleotide polymorphisms in the instrument. IVW: Inverse variance weighted. MR: Mendelian randomization. MR-PRESSO: Mendelian Randomization Pleiotropy RESidual Sum and Outlier. OR: Odds ratio. CI: confidence interval. Beta: MR effect estimate. Se: standard error of MR effect estimate. P: P-value. PD: Parkinson’s Disease. Describing PD risk results using OR (95% CI)

## Discussion

In this analysis, we have demonstrated the potential protective effect of T1DM on PD risk. Our MVMR analysis suggests that this observed causal relationship may be driven by drug-related features of specific anti-diabetic medications. We thoroughly examined the data using various sensitivity methods in the MR analysis and found no significant pleiotropy or heterogeneity. Moreover, no evidence supports a causal relationship between genetically predicted T2DM and PD. To delve deeper into the topic, we further analyzed the causal relationship between glycemic traits and PD. However, the results of this analysis do not support a causal relationship between the two.

There has been a long-standing controversy regarding the association between DM and PD. Epidemiological evidence suggests an association between DM and PD, but the results are inconsistent, ranging from significant negative correlations to significant positive correlations [35–40]. Biological evidence demonstrates that both conditions are characterized by abnormal protein accumulation, lysosomal and mitochondrial dysfunction, and chronic systemic inflammation [41, 42]. Moreover, hypoinsulinemia in T1DM patients or insulin resistance (IR) in T2D patients leads to hyperglycemia, exposing neurons to increased metabolic stress, neuronal dysfunction, and death, thereby directly contributing to the development of PD [43]. Furthermore, several anti-diabetic drugs have been shown to have anti-PD effects, such as DPP-4 inhibitors and GLP-1 receptor agonists [44–46]. However, these studies often have relatively small sample sizes, which may introduce confounding, selection bias, and reverse causality, further limiting the interpretability of the results [47]. Additionally, case-control studies do not adequately address the temporal relationship between diabetes and PD since they rely on retrospective data and often fail to specify the time window for exposure assessment. Although large-scale prospective studies hold promise in overcoming these limitations, conducting such research requires significant human, financial, and time resources.

Although clinical trials have various limitations, early identification of risk factors for PD is crucial. Early intervention targeting relevant risk factors is currently the most effective approach to delay or prevent the onset of PD [48]. However, there is currently no effective cure once PD occurs. Compared to traditional epidemiology, MR analysis reveals the causal relationship between DM and PD cost-effectively, reducing confounding biases in epidemiological studies, including reverse causation [49, 50]. Three Mendelian randomization studies have recently been reported, investigating the causal inference of DM on PD in different populations. Chohan et al.‘s MR study on the European population reveals that genetically predicted T2DM leads to an increased risk and faster progression of PD, particularly in motor impairment [51]. Park et al.‘s MR study based on the Korean (East Asian) population suggests no evidence of a causal association between T2DM and PD. The authors explain this seemingly contradictory result as being due to a small sample size and ethnic differences [51, 52]. Additionally, Senkevich et al.‘s MR study on the European population suggests a potential protective association between genetically predicted T1DM and the risk and progression of PD, possibly driven by latent pleiotropy [53].

There is ongoing controversy regarding the relationship between DM and PD; given the complex association and significant clinical implications between the two, it is imperative to robustly replicate this association in larger GWAS study cohorts and explore potential underlying mechanisms. Consistent with the findings of Senkevich et al., our results confirm the causal relationship between T1DM and reduced risk of PD, and we further discovered that the use of anti-diabetic medications may mediate this causal relationship. Some traditional epidemiological approaches have also reported a lower risk of PD incidence in DM patients [37, 38, 54]. It has been reported that long-term use of anti-diabetic medications such as GLP-2 receptor agonists and DPP1 inhibitors may potentially reduce the risk of PD [45]. In recent years, an increasing body of research evidence supports the potential of anti-diabetic medications in reducing the risk of PD [55]. Using commonly used anti-diabetic drugs targeting the insulin signaling pathway has induced neuroprotective effects in preclinical studies and clinical trials. A longitudinal study of 5,528 veterans with T2DM showed that treatment with metformin for more than four years can reduce the risk of AD and PD [56]. The neuroprotective effect of metformin is mediated through the regulation of AMP-activated protein kinase (AMPK) activity, which modulates several critical cellular processes such as autophagy, cell growth, and mitochondrial function, as well as inhibiting microglial activation and inflammation [57–60]. Some studies have explored the neuroprotective potential of intranasal insulin. Preclinical data indicate that intranasal delivery of recombinant human insulin can reach deep brain structures, including the hippocampus and nigrostriatal pathway [61]. The study by Novak et al. showed that intranasal short-acting (regular) insulin treatment improved motor performance and function compared to placebo, resulting in lower disability scores (HY scale) and improved UPDRS motor scores compared to placebo [46].

Furthermore, other drugs, such as glucagon-like peptide 1 (GLP-1) agonists, can provide neuroprotection. Liraglutide and lixisenatide, both GLP-1 analogs, have been shown to induce neuroprotection in PD animal models [62]. These drugs can cross the blood-brain barrier (BBB), enhance hippocampal neurogenesis, and increase brain-derived neurotrophic factor (BDNF) expression, promoting neuroprotection in AD and PD [63, 64].

Our study highlights the potential protective effect of genetic prediction of T1DM on PD, suggesting that anti-diabetic drugs may play a crucial role in reducing PD risk. However, the exact mechanism underlying this protective effect remains unclear. Therefore, it is necessary to gather further direct evidence to validate our findings and develop effective PD prevention and management strategies.

We want to acknowledge certain limitations in our study. Firstly, it is essential to note that the associations observed through MR analysis do not provide information about temporal patterns but rather reflect lifelong effects on specific risk factors. Secondly, the sample size used for analyzing PD progression (UPDRS3/MMSE/MOCA) is relatively small, which may reduce the analytical power and potentially lead to false-negative results. Conducting larger-scale MR analyses will be essential to ensure the robustness of our findings. Additionally, it should be considered that genetic variations associated with T1DM may be correlated with multiple factors, which could represent alternative pathways through which these genetic variations influence PD. This potential horizontal pleiotropy should be taken into account when interpreting our results. Lastly, it is worth mentioning that our study primarily focuses on individuals of European ancestry. Further research is needed to determine whether our findings can be generalized to other ethnicities.

## Conclusion

In summary, our study discovered a direct causal relationship between genetic predictions of T1DM and a decreased risk of PD in individuals of European ancestry. Moreover, there is indirect evidence indicating that anti-diabetic drugs may mediate the protective effect of T1DM against PD. However, further research is needed to fully understand the mechanisms by which anti-diabetic drugs exert their anti-PD effects and to identify potential therapeutic targets.

### Electronic supplementary material

Below is the link to the electronic supplementary material.

Supplementary Material 1

## Data Availability

The summary statistics analyzed in the study are included in the article. MAGIC investigators have contributed data on glycaemic traits and have been downloaded from www.magicinvestigators.org
