# Supplementary material for: Causal relationship between diabetes mellitus, glycemic traits and Parkinson’s disease: a multivariable mendelian randomization analysis
Source: Diabetol Metab Syndr. 2024 Mar 5;16:59. doi: 10.1186/s13098-024-01299-8 (PMC10913216; doi:10.1186/s13098-024-01299-8)
Supplement: Supplementary file 1 — Supplementary Material 1 [file 13098_2024_1299_MOESM1_ESM.docx]

**Supplementary Information**

**Causal Relationship between Diabetes Mellitus, Glycemic Profile and Parkinson's Disease: a Multivariable Mendelian Randomization Analysis**

**Qitong Wang ^1, #^, Benchi Cai ^1, #^, Lifan Zhong ^1^, Jitrawadee Intirach ^1^, Tao Chen ^1, 2,^ ***

*1 Hainan General Hospital, Hainan Afliated Hospital of Hainan Medical University, Haikou, Hainan 570311, China*

*2 Hainan Provincial Bureau of Disease Prevention and Control, Haikou 570100, China*

**Contents**

**Table S1** Instrumental variables for Mendelian randomization(MR) analysis

**Table S2** Phenotypes significantly associated with instrumental variables of Diabetes Mellitus and Glycemic Traits identified by PhenoScanner.

**Table S3** Results of the MR analysis and Sensitivity analysis in forward MR

**Table S4** MR assessment for the significant results of reverse MR analyses.

**Table S5** Multivariable MR results after adjusting for the Anti-diabetic drug.

**Table S1** Instrumental variables for Mendelian randomization(MR) analysis

| **SNP** | **EA** | **OA** | **EAF** | **BETA** | **SE** | **P** | **R2** | **F-statistic** |
| --- | --- | --- | --- | --- | --- | --- | --- | --- |
| **Type 1 diabetes (T1DM)** | | | | | | | | |
| rs41295159 | G | C | 0.00895 | -0.699552 | 0.090252 | 9.11E-15 | 2.13E-05 | 11.0800443 |
| rs12665124 | T | G | 0.0132 | -0.494982 | 0.070847 | 2.82E-12 | 2.71E-05 | 14.11486948 |
| rs78325861 | G | C | 0.039 | -0.282082 | 0.042195 | 2.31E-11 | 4.55E-05 | 23.69939941 |
| rs34536443 | C | G | 0.0427 | -0.385331 | 0.038519 | 1.47E-23 | 4.99E-05 | 25.96111421 |
| rs112647257 | T | A | 0.0527 | -0.721883 | 0.038225 | 1.51E-79 | 5.03E-05 | 26.16078896 |
| rs55969931 | G | T | 0.0421 | -0.459986 | 0.038112 | 1.53E-33 | 5.04E-05 | 26.23835428 |
| rs12257077 | T | C | 0.0319 | 0.231245 | 0.036955 | 3.91E-10 | 5.20E-05 | 27.0598338 |
| rs7776597 | G | A | 0.959 | 0.244413 | 0.036372 | 1.82E-11 | 5.28E-05 | 27.49357083 |
| rs114378220 | T | C | 0.0703 | 0.177902 | 0.030444 | 5.11E-09 | 6.31E-05 | 32.84706865 |
| rs72838204 | T | C | 0.0623 | 0.376892 | 0.028072 | 4.26E-41 | 6.84E-05 | 35.62254767 |
| rs61839660 | T | C | 0.0851 | -0.357441 | 0.026 | 5.25E-43 | 7.39E-05 | 38.4613907 |
| rs55993634 | G | C | 0.0849 | 0.219371 | 0.024379 | 2.29E-19 | 7.88E-05 | 41.01875213 |
| rs34593439 | A | G | 0.108 | -0.218071 | 0.024119 | 1.54E-19 | 7.96E-05 | 41.46092948 |
| rs574384 | A | C | 0.895 | -0.133602 | 0.023876 | 2.20E-08 | 8.04E-05 | 41.88290158 |
| rs17623914 | C | T | 0.0997 | -0.134887 | 0.023381 | 7.97E-09 | 8.22E-05 | 42.76960601 |
| rs2523679 | T | C | 0.0964 | 0.711041 | 0.022941 | 1.00E-200 | 8.37E-05 | 43.58991143 |
| rs2493411 | C | T | 0.132 | 0.127063 | 0.022335 | 1.28E-08 | 8.60E-05 | 44.77260614 |
| rs12128789 | C | T | 0.132 | 0.126969 | 0.021535 | 3.73E-09 | 8.92E-05 | 46.43585596 |
| rs231972 | C | A | 0.118 | 0.170939 | 0.021081 | 5.12E-16 | 9.11E-05 | 47.43589764 |
| rs74999184 | T | C | 0.719 | 0.948382 | 0.021037 | 1.00E-200 | 9.13E-05 | 47.53511233 |
| rs6679677 | A | C | 0.114 | 0.64172 | 0.020938 | 1.00E-200 | 9.17E-05 | 47.75987 |
| rs113374757 | T | C | 0.163 | -0.171277 | 0.020769 | 1.63E-16 | 9.25E-05 | 48.14849815 |
| rs7110099 | G | A | 0.787 | 0.662564 | 0.020187 | 1.00E-200 | 9.51E-05 | 49.53664032 |
| rs114278107 | G | T | 0.174 | -0.146158 | 0.019861 | 1.85E-13 | 9.67E-05 | 50.34973859 |
| rs3024493 | A | C | 0.154 | -0.163855 | 0.019641 | 7.26E-17 | 9.78E-05 | 50.91370898 |
| rs12644686 | G | C | 0.194 | -0.10775 | 0.01932 | 2.44E-08 | 9.94E-05 | 51.75963551 |
| rs3802214 | C | T | 0.799 | -0.106609 | 0.01923 | 2.96E-08 | 9.99E-05 | 52.0018803 |
| rs6434435 | A | G | 0.16 | -0.122856 | 0.019089 | 1.23E-10 | 1.01E-04 | 52.38598974 |
| rs2611211 | T | C | 0.824 | -0.143854 | 0.018689 | 1.39E-14 | 1.03E-04 | 53.50720521 |
| rs7237497 | C | T | 0.839 | -0.220466 | 0.018635 | 2.71E-32 | 1.03E-04 | 53.66225694 |
| rs61759532 | T | C | 0.235 | 0.118379 | 0.018587 | 1.91E-10 | 1.03E-04 | 53.80083704 |
| rs6908626 | T | G | 0.166 | 0.202923 | 0.01852 | 6.14E-28 | 1.04E-04 | 53.9954729 |
| rs202535 | A | C | 0.828 | -0.141437 | 0.018453 | 1.79E-14 | 1.04E-04 | 54.19152214 |
| rs57209021 | T | C | 0.226 | 0.100693 | 0.018297 | 3.73E-08 | 1.05E-04 | 54.6535584 |
| rs13018977 | A | T | 0.225 | 0.100335 | 0.017882 | 2.01E-08 | 1.07E-04 | 55.92194151 |
| rs1881146 | T | A | 0.311 | -0.09517 | 0.017407 | 4.57E-08 | 1.10E-04 | 57.44793233 |
| rs55893453 | G | A | 0.202 | 0.094653 | 0.01732 | 4.63E-08 | 1.11E-04 | 57.73649874 |
| rs1947178 | G | A | 0.792 | -0.103267 | 0.017134 | 1.67E-09 | 1.12E-04 | 58.36326358 |
| rs855330 | C | T | 0.259 | 0.111208 | 0.016916 | 4.89E-11 | 1.14E-04 | 59.11540306 |
| rs17323934 | G | C | 0.223 | -0.129663 | 0.016818 | 1.26E-14 | 1.14E-04 | 59.45987383 |
| rs4548024 | C | T | 0.234 | -0.095737 | 0.016703 | 9.95E-09 | 1.15E-04 | 59.86925451 |
| rs7795896 | T | C | 0.692 | -0.135435 | 0.016416 | 1.58E-16 | 1.17E-04 | 60.91594531 |
| rs2395471 | A | G | 0.366 | -0.522124 | 0.016351 | 1.00E-200 | 1.17E-04 | 61.15810398 |
| rs7068821 | T | G | 0.251 | -0.165103 | 0.016333 | 5.07E-24 | 1.18E-04 | 61.22550408 |
| rs9517712 | C | T | 0.741 | -0.102063 | 0.015805 | 1.06E-10 | 1.22E-04 | 63.27087366 |
| rs10801128 | G | A | 0.717 | 0.096073 | 0.015681 | 8.98E-10 | 1.22E-04 | 63.77119815 |
| rs1008438 | C | A | 0.409 | 0.503981 | 0.015469 | 1.00E-200 | 1.24E-04 | 64.64517151 |
| rs4490209 | G | C | 0.36 | -0.084522 | 0.015457 | 4.55E-08 | 1.24E-04 | 64.69535862 |
| rs10224046 | G | T | 0.324 | 0.085811 | 0.015436 | 2.71E-08 | 1.24E-04 | 64.78337381 |
| rs1350275 | G | T | 0.698 | -0.093665 | 0.015283 | 8.86E-10 | 1.26E-04 | 65.43192816 |
| rs7668577 | C | A | 0.312 | 0.093652 | 0.015203 | 7.26E-10 | 1.26E-04 | 65.77623878 |
| rs12927355 | T | C | 0.316 | -0.203881 | 0.015188 | 4.41E-41 | 1.26E-04 | 65.84120083 |
| rs8046043 | C | G | 0.392 | -0.084587 | 0.015175 | 2.49E-08 | 1.27E-04 | 65.89760515 |
| rs238265 | G | T | 0.695 | -0.090825 | 0.015158 | 2.08E-09 | 1.27E-04 | 65.97151063 |
| rs663743 | A | G | 0.349 | -0.099964 | 0.015092 | 3.50E-11 | 1.27E-04 | 66.26001578 |
| rs12742756 | G | A | 0.428 | -0.083107 | 0.015077 | 3.54E-08 | 1.27E-04 | 66.3259374 |
| rs1808094 | C | T | 0.524 | -0.113651 | 0.0149 | 2.40E-14 | 1.29E-04 | 67.11383612 |
| rs9385401 | T | C | 0.454 | 0.12042 | 0.014827 | 4.59E-16 | 1.30E-04 | 67.44426776 |
| rs17106304 | G | C | 0.656 | 0.115351 | 0.014812 | 6.83E-15 | 1.30E-04 | 67.51256806 |
| rs13147049 | G | A | 0.641 | -0.109522 | 0.014689 | 8.92E-14 | 1.31E-04 | 68.07789217 |
| rs13259300 | C | A | 0.598 | -0.092191 | 0.014669 | 3.28E-10 | 1.31E-04 | 68.1707109 |
| rs56994090 | C | T | 0.431 | -0.134255 | 0.014594 | 3.60E-20 | 1.32E-04 | 68.52104688 |
| rs1701704 | G | T | 0.339 | 0.244048 | 0.014558 | 4.52E-63 | 1.32E-04 | 68.69049032 |
| rs2111485 | G | A | 0.604 | 0.127631 | 0.014455 | 1.05E-18 | 1.33E-04 | 69.17994868 |
| rs722988 | C | T | 0.353 | 0.082649 | 0.014412 | 9.78E-09 | 1.33E-04 | 69.38635568 |
| rs11203203 | A | G | 0.349 | 0.143803 | 0.014405 | 1.81E-23 | 1.33E-04 | 69.42007346 |
| rs12464462 | G | A | 0.41 | -0.087955 | 0.014341 | 8.61E-10 | 1.34E-04 | 69.72987645 |
| rs2543537 | T | C | 0.46 | -0.083441 | 0.014316 | 5.59E-09 | 1.34E-04 | 69.85164558 |
| rs607703 | T | C | 0.484 | 0.092015 | 0.01428 | 1.17E-10 | 1.35E-04 | 70.02774217 |
| rs4820827 | C | T | 0.621 | -0.129657 | 0.014267 | 1.01E-19 | 1.35E-04 | 70.091551 |
| rs3087243 | A | G | 0.422 | -0.19913 | 0.014202 | 1.16E-44 | 1.35E-04 | 70.41234743 |
| rs1574285 | T | G | 0.591 | -0.126548 | 0.014171 | 4.27E-19 | 1.36E-04 | 70.56637909 |
| rs229527 | A | C | 0.416 | 0.104077 | 0.014138 | 1.82E-13 | 1.36E-04 | 70.73109055 |
| rs1050979 | G | A | 0.515 | 0.106196 | 0.014129 | 5.65E-14 | 1.36E-04 | 70.77614538 |
| rs2188962 | T | C | 0.404 | 0.07946 | 0.014096 | 1.73E-08 | 1.36E-04 | 70.94183869 |
| rs10751776 | C | A | 0.51 | 0.078145 | 0.01405 | 2.67E-08 | 1.37E-04 | 71.17410378 |
| rs2303137 | T | A | 0.442 | -0.081513 | 0.014025 | 6.17E-09 | 1.37E-04 | 71.30097384 |
| rs7936434 | C | G | 0.464 | 0.076923 | 0.013959 | 3.58E-08 | 1.38E-04 | 71.63809429 |
| **Type 2 diabetes (T2DM)** | | | | | | | | |
| rs116425039 | A | G | 0.012 | -0.272 | 0.0348 | 5.08E-15 | 3.08E-05 | 28.73557065 |
| rs76895963 | T | G | 0.9801 | 0.4826 | 0.0275 | 9.12E-69 | 3.89E-05 | 36.36355849 |
| rs139688524 | T | C | 0.0226 | 0.1739 | 0.0216 | 7.47E-16 | 4.96E-05 | 46.29619716 |
| rs141521721 | A | C | 0.0233 | 0.1212 | 0.0214 | 1.39E-08 | 5.00E-05 | 46.7288719 |
| rs1800961 | T | C | 0.0349 | 0.1602 | 0.0175 | 5.1E-20 | 6.12E-05 | 57.14273478 |
| rs145678014 | T | G | 0.0426 | -0.1048 | 0.0163 | 1.4E-10 | 6.57E-05 | 61.34956188 |
| rs62107261 | T | C | 0.9541 | 0.1019 | 0.0161 | 2.62E-10 | 6.65E-05 | 62.11166824 |
| rs56187241 | T | C | 0.0451 | 0.1014 | 0.0157 | 1.07E-10 | 6.82E-05 | 63.69413112 |
| rs115505614 | T | C | 0.0502 | 0.1657 | 0.0149 | 7.58E-29 | 7.19E-05 | 67.11395024 |
| rs62271373 | A | T | 0.0547 | 0.0882 | 0.0144 | 1.03E-09 | 7.43E-05 | 69.44429574 |
| rs11063029 | T | C | 0.0581 | 0.0858 | 0.0138 | 5.36E-10 | 7.76E-05 | 72.46361294 |
| rs7568172 | A | G | 0.0614 | -0.0827 | 0.0135 | 9.19E-10 | 7.93E-05 | 74.07391545 |
| rs17791513 | A | G | 0.9323 | 0.1016 | 0.0132 | 1.35E-14 | 8.11E-05 | 75.75741353 |
| rs77864822 | A | G | 0.9295 | 0.0753 | 0.0129 | 5.01E-09 | 8.30E-05 | 77.51921385 |
| rs17772814 | A | G | 0.0857 | -0.0746 | 0.0125 | 2.13E-09 | 8.56E-05 | 79.99982869 |
| rs2062213 | C | G | 0.924 | -0.0743 | 0.012 | 6.8E-10 | 8.92E-05 | 83.33315488 |
| rs739846 | A | G | 0.0761 | 0.0877 | 0.0119 | 2.02E-13 | 9.00E-05 | 84.0334335 |
| rs2023681 | A | G | 0.087 | -0.0826 | 0.0115 | 7.4E-13 | 9.31E-05 | 86.95633553 |
| rs12140153 | T | G | 0.0953 | -0.0645 | 0.0113 | 1.17E-08 | 9.47E-05 | 88.49538572 |
| rs6885132 | C | G | 0.9036 | 0.077 | 0.011 | 2.49E-12 | 9.73E-05 | 90.90889624 |
| rs672271 | T | C | 0.9062 | -0.06 | 0.011 | 4.83E-08 | 9.73E-05 | 90.90889624 |
| rs17030845 | T | C | 0.1007 | -0.1191 | 0.0108 | 2.35E-28 | 9.91E-05 | 92.59239432 |
| rs72802358 | C | G | 0.1032 | -0.1135 | 0.0107 | 2.22E-26 | 1.00E-04 | 93.45774379 |
| rs9379084 | A | G | 0.1121 | -0.0994 | 0.0106 | 5.48E-21 | 1.01E-04 | 94.33942062 |
| rs2258238 | A | T | 0.8963 | -0.102 | 0.0106 | 5.14E-22 | 1.01E-04 | 94.33942062 |
| rs59147390 | T | C | 0.8922 | 0.0648 | 0.0105 | 6.06E-10 | 1.02E-04 | 95.2378913 |
| rs11680058 | A | G | 0.8648 | 0.0581 | 0.0104 | 2.08E-08 | 1.03E-04 | 96.15364025 |
| rs17744783 | A | T | 0.1073 | 0.0577 | 0.0103 | 1.87E-08 | 1.04E-04 | 97.08717074 |
| rs58432198 | T | C | 0.1172 | -0.0636 | 0.0103 | 5.71E-10 | 1.04E-04 | 97.08717074 |
| rs1493694 | T | C | 0.1096 | 0.08 | 0.0102 | 3.35E-15 | 1.05E-04 | 98.03900575 |
| rs34715063 | T | C | 0.8778 | -0.0772 | 0.0099 | 8.4E-15 | 1.08E-04 | 101.0098847 |
| rs11709077 | A | G | 0.1264 | -0.104 | 0.0097 | 1.26E-26 | 1.10E-04 | 103.0925627 |
| rs2107133 | A | G | 0.8727 | 0.0643 | 0.0097 | 4.02E-11 | 1.10E-04 | 103.0925627 |
| rs6444809 | T | C | 0.8723 | 0.0544 | 0.0096 | 1.63E-08 | 1.12E-04 | 104.1664436 |
| rs4709746 | T | C | 0.1313 | -0.0567 | 0.0096 | 3.95E-09 | 1.12E-04 | 104.1664436 |
| rs7325671 | T | C | 0.1264 | 0.0545 | 0.0096 | 1.53E-08 | 1.12E-04 | 104.1664436 |
| rs9665898 | T | C | 0.1361 | -0.0525 | 0.0094 | 2.53E-08 | 1.14E-04 | 106.3827509 |
| rs58642235 | T | C | 0.8612 | -0.0566 | 0.0094 | 1.9E-09 | 1.14E-04 | 106.3827509 |
| rs7313918 | T | C | 0.8635 | -0.0559 | 0.0094 | 2.99E-09 | 1.14E-04 | 106.3827509 |
| rs2290202 | T | G | 0.1393 | 0.0666 | 0.0092 | 4.89E-13 | 1.16E-04 | 108.6954194 |
| rs12920022 | A | T | 0.1582 | 0.0528 | 0.0092 | 0.00000001 | 1.16E-04 | 108.6954194 |
| rs2292662 | T | C | 0.16 | -0.0645 | 0.0088 | 2.24E-13 | 1.22E-04 | 113.6361203 |
| rs13022337 | A | G | 0.1715 | -0.0519 | 0.0088 | 3.61E-09 | 1.22E-04 | 113.6361203 |
| rs77464186 | A | C | 0.8357 | 0.1002 | 0.0087 | 9.3E-31 | 1.23E-04 | 114.9422826 |
| rs601945 | A | G | 0.8259 | -0.0849 | 0.0086 | 4.66E-23 | 1.24E-04 | 116.2788208 |
| rs10811660 | A | G | 0.1704 | -0.1598 | 0.0086 | 2.54E-77 | 1.24E-04 | 116.2788208 |
| rs576674 | A | G | 0.8313 | -0.0538 | 0.0086 | 3.7E-10 | 1.24E-04 | 116.2788208 |
| rs6600191 | T | C | 0.8246 | 0.0587 | 0.0085 | 4.47E-12 | 1.26E-04 | 117.6468069 |
| rs745805 | A | T | 0.82 | 0.0632 | 0.0084 | 4.52E-14 | 1.27E-04 | 119.0473641 |
| rs11496066 | T | C | 0.8173 | 0.0508 | 0.0083 | 8.17E-10 | 1.29E-04 | 120.4816697 |
| rs17168486 | T | C | 0.1802 | 0.0672 | 0.0083 | 4.5E-16 | 1.29E-04 | 120.4816697 |
| rs3094682 | A | C | 0.1926 | -0.0619 | 0.0082 | 3.47E-14 | 1.31E-04 | 121.9509584 |
| rs3798519 | A | C | 0.811 | -0.0616 | 0.0082 | 4.6E-14 | 1.31E-04 | 121.9509584 |
| rs1517037 | T | C | 0.1935 | -0.0451 | 0.0082 | 3.35E-08 | 1.31E-04 | 121.9509584 |
| rs7966976 | A | G | 0.1964 | -0.0751 | 0.0081 | 1.22E-20 | 1.32E-04 | 123.4565258 |
| rs61676547 | C | G | 0.1937 | 0.0525 | 0.0081 | 7.42E-11 | 1.32E-04 | 123.4565258 |
| rs1783541 | T | C | 0.201 | 0.0608 | 0.0081 | 4.65E-14 | 1.32E-04 | 123.4565258 |
| rs3768321 | T | G | 0.1993 | 0.084 | 0.008 | 4.75E-26 | 1.34E-04 | 124.9997323 |
| rs28533815 | T | C | 0.2507 | -0.0748 | 0.008 | 5.45E-21 | 1.34E-04 | 124.9997323 |
| rs67232546 | T | C | 0.2047 | 0.0531 | 0.008 | 2.51E-11 | 1.34E-04 | 124.9997323 |
| rs490689 | A | G | 0.2002 | 0.0543 | 0.008 | 8.86E-12 | 1.34E-04 | 124.9997323 |
| rs1496653 | A | G | 0.7887 | 0.0665 | 0.0079 | 2.49E-17 | 1.36E-04 | 126.5820074 |
| rs11063069 | A | G | 0.7868 | -0.056 | 0.0079 | 9.94E-13 | 1.36E-04 | 126.5820074 |
| rs11708067 | A | G | 0.775 | 0.0882 | 0.0077 | 5.05E-30 | 1.39E-04 | 129.8698518 |
| rs1431841 | T | G | 0.2113 | 0.0429 | 0.0077 | 3.08E-08 | 1.39E-04 | 129.8698518 |
| rs17122772 | C | G | 0.7736 | -0.0428 | 0.0077 | 3.31E-08 | 1.39E-04 | 129.8698518 |
| rs11257655 | T | C | 0.2169 | 0.0859 | 0.0077 | 1.46E-28 | 1.39E-04 | 129.8698518 |
| rs11688682 | C | G | 0.2721 | -0.0581 | 0.0076 | 2.93E-14 | 1.41E-04 | 131.5786656 |
| rs1117610 | A | T | 0.7737 | -0.0423 | 0.0076 | 3.13E-08 | 1.41E-04 | 131.5786656 |
| rs8008910 | A | G | 0.2192 | 0.0554 | 0.0076 | 4.22E-13 | 1.41E-04 | 131.5786656 |
| rs9304665 | A | T | 0.7595 | 0.0418 | 0.0076 | 4.53E-08 | 1.41E-04 | 131.5786656 |
| rs4865436 | C | G | 0.7004 | -0.049 | 0.0075 | 8.04E-11 | 1.43E-04 | 133.3330478 |
| rs11759026 | A | G | 0.768 | -0.066 | 0.0075 | 2.04E-18 | 1.43E-04 | 133.3330478 |
| rs508419 | A | G | 0.2356 | -0.0811 | 0.0075 | 5.43E-27 | 1.43E-04 | 133.3330478 |
| rs35011184 | A | G | 0.2306 | 0.2822 | 0.0075 | 1E-200 | 1.43E-04 | 133.3330478 |
| rs2080385 | T | G | 0.2489 | -0.0551 | 0.0074 | 1.24E-13 | 1.45E-04 | 135.1348458 |
| rs878521 | A | G | 0.2434 | 0.0618 | 0.0074 | 9.31E-17 | 1.45E-04 | 135.1348458 |
| rs1426371 | A | G | 0.2659 | -0.0517 | 0.0073 | 1.74E-12 | 1.47E-04 | 136.986008 |
| rs11842871 | T | G | 0.2669 | -0.04 | 0.0073 | 4.83E-08 | 1.47E-04 | 136.986008 |
| rs6687271 | A | C | 0.7521 | -0.0402 | 0.0073 | 4.14E-08 | 1.47E-04 | 136.986008 |
| rs459193 | A | G | 0.2594 | -0.0722 | 0.0073 | 6.78E-23 | 1.47E-04 | 136.986008 |
| rs7572970 | A | G | 0.2659 | -0.0447 | 0.0072 | 6.12E-10 | 1.49E-04 | 138.8885915 |
| rs55653563 | A | C | 0.7333 | 0.0435 | 0.0072 | 1.73E-09 | 1.49E-04 | 138.8885915 |
| rs11048456 | T | C | 0.7407 | -0.0454 | 0.0072 | 3.3E-10 | 1.49E-04 | 138.8885915 |
| rs9563615 | A | T | 0.7092 | 0.0408 | 0.0072 | 1.63E-08 | 1.49E-04 | 138.8885915 |
| rs8097210 | T | G | 0.7325 | -0.0537 | 0.0072 | 1.06E-13 | 1.49E-04 | 138.8885915 |
| rs3786900 | A | G | 0.7307 | 0.0434 | 0.0072 | 1.89E-09 | 1.49E-04 | 138.8885915 |
| rs1561927 | T | C | 0.7328 | -0.042 | 0.0072 | 6.12E-09 | 1.49E-04 | 138.8885915 |
| rs12680692 | A | T | 0.3206 | 0.0413 | 0.0071 | 6.6E-09 | 1.51E-04 | 140.8447688 |
| rs10830963 | C | G | 0.7208 | -0.101 | 0.0071 | 1.12E-45 | 1.51E-04 | 140.8447688 |
| rs1359790 | A | G | 0.2791 | -0.0817 | 0.0071 | 1.76E-30 | 1.51E-04 | 140.8447688 |
| rs7667864 | A | C | 0.2838 | -0.0402 | 0.0071 | 1.64E-08 | 1.51E-04 | 140.8447688 |
| rs9368222 | A | C | 0.2717 | 0.1379 | 0.0071 | 1.42E-83 | 1.51E-04 | 140.8447688 |
| rs10750397 | A | G | 0.2829 | 0.0394 | 0.0071 | 3.13E-08 | 1.51E-04 | 140.8447688 |
| rs5758223 | A | G | 0.7134 | 0.0401 | 0.0071 | 1.78E-08 | 1.51E-04 | 140.8447688 |
| rs4368494 | A | G | 0.2976 | -0.054 | 0.007 | 1.39E-14 | 1.53E-04 | 142.8568369 |
| rs4688760 | T | C | 0.6828 | 0.042 | 0.007 | 2.14E-09 | 1.53E-04 | 142.8568369 |
| rs8192675 | T | C | 0.7121 | 0.0659 | 0.007 | 5.77E-21 | 1.53E-04 | 142.8568369 |
| rs1061810 | A | C | 0.2867 | 0.0502 | 0.007 | 8.31E-13 | 1.53E-04 | 142.8568369 |
| rs2277536 | T | C | 0.7011 | -0.0429 | 0.007 | 9.63E-10 | 1.53E-04 | 142.8568369 |
| rs2925979 | T | C | 0.2987 | 0.0546 | 0.007 | 7.07E-15 | 1.53E-04 | 142.8568369 |
| rs7669833 | A | T | 0.2951 | -0.0572 | 0.007 | 3.52E-16 | 1.53E-04 | 142.8568369 |
| rs7732130 | A | G | 0.6992 | -0.0606 | 0.007 | 5.69E-18 | 1.53E-04 | 142.8568369 |
| rs2767036 | A | C | 0.7086 | -0.0389 | 0.007 | 2.94E-08 | 1.53E-04 | 142.8568369 |
| rs35318451 | A | G | 0.331 | 0.0478 | 0.007 | 9.5E-12 | 1.53E-04 | 142.8568369 |
| rs12910361 | A | G | 0.2926 | -0.0814 | 0.007 | 3.95E-31 | 1.53E-04 | 142.8568369 |
| rs2351707 | T | C | 0.7178 | -0.0644 | 0.007 | 4.3E-20 | 1.53E-04 | 142.8568369 |
| rs1562396 | A | G | 0.6796 | -0.0555 | 0.0069 | 9.64E-16 | 1.55E-04 | 144.9272259 |
| rs2820441 | A | C | 0.6893 | 0.0556 | 0.0069 | 8.56E-16 | 1.55E-04 | 144.9272259 |
| rs10516495 | A | T | 0.3128 | -0.0418 | 0.0069 | 1.46E-09 | 1.55E-04 | 144.9272259 |
| rs702634 | A | G | 0.689 | 0.0503 | 0.0069 | 3.37E-13 | 1.55E-04 | 144.9272259 |
| rs4977213 | T | C | 0.6281 | -0.0507 | 0.0069 | 2.19E-13 | 1.55E-04 | 144.9272259 |
| rs56348580 | C | G | 0.3082 | -0.0617 | 0.0069 | 4.31E-19 | 1.55E-04 | 144.9272259 |
| rs35895680 | A | C | 0.323 | -0.0554 | 0.0069 | 1.08E-15 | 1.55E-04 | 144.9272259 |
| rs3802177 | A | G | 0.3154 | -0.1077 | 0.0069 | 9.16E-55 | 1.55E-04 | 144.9272259 |
| rs4925109 | A | G | 0.3177 | 0.0476 | 0.0069 | 5.65E-12 | 1.55E-04 | 144.9272259 |
| rs7633675 | T | G | 0.6837 | -0.1085 | 0.0068 | 3.21E-57 | 1.57E-04 | 147.0585086 |
| rs1007090 | T | C | 0.3316 | -0.044 | 0.0068 | 1.01E-10 | 1.57E-04 | 147.0585086 |
| rs28663084 | A | G | 0.673 | -0.0376 | 0.0068 | 0.000000033 | 1.57E-04 | 147.0585086 |
| rs1665901 | A | T | 0.6576 | 0.0398 | 0.0068 | 4.97E-09 | 1.57E-04 | 147.0585086 |
| rs1412234 | T | C | 0.6739 | -0.0393 | 0.0068 | 7.71E-09 | 1.57E-04 | 147.0585086 |
| rs505922 | T | C | 0.6634 | -0.0473 | 0.0068 | 3.65E-12 | 1.57E-04 | 147.0585086 |
| rs4929965 | A | G | 0.3813 | 0.0668 | 0.0068 | 9.67E-23 | 1.57E-04 | 147.0585086 |
| rs320369 | A | G | 0.3232 | 0.0372 | 0.0068 | 0.000000046 | 1.57E-04 | 147.0585086 |
| rs3019208 | A | G | 0.6847 | 0.0397 | 0.0068 | 5.43E-09 | 1.57E-04 | 147.0585086 |
| rs8071043 | T | C | 0.6738 | -0.0523 | 0.0068 | 1.53E-14 | 1.57E-04 | 147.0585086 |
| rs79920718 | A | G | 0.3385 | 0.0389 | 0.0067 | 6.43E-09 | 1.60E-04 | 149.2534117 |
| rs6459737 | A | G | 0.338 | -0.0583 | 0.0067 | 3.31E-18 | 1.60E-04 | 149.2534117 |
| rs348330 | A | G | 0.6399 | -0.0492 | 0.0067 | 2.1E-13 | 1.60E-04 | 149.2534117 |
| rs13130484 | T | C | 0.4296 | 0.0435 | 0.0067 | 8.49E-11 | 1.60E-04 | 149.2534117 |
| rs10974438 | A | C | 0.6416 | -0.0514 | 0.0067 | 1.71E-14 | 1.60E-04 | 149.2534117 |
| rs231360 | T | C | 0.3963 | 0.0576 | 0.0067 | 8.27E-18 | 1.60E-04 | 149.2534117 |
| rs1468906 | A | G | 0.6442 | 0.0388 | 0.0067 | 7.03E-09 | 1.60E-04 | 149.2534117 |
| rs35777422 | A | G | 0.3559 | -0.0381 | 0.0067 | 0.000000013 | 1.60E-04 | 149.2534117 |
| rs3783394 | A | G | 0.3477 | -0.038 | 0.0067 | 1.42E-08 | 1.60E-04 | 149.2534117 |
| rs10908278 | A | T | 0.5174 | -0.0749 | 0.0067 | 5.25E-29 | 1.60E-04 | 149.2534117 |
| rs10937721 | C | G | 0.5893 | 0.085 | 0.0066 | 5.39E-38 | 1.62E-04 | 151.5148271 |
| rs5215 | T | C | 0.6272 | -0.0706 | 0.0066 | 9.85E-27 | 1.62E-04 | 151.5148271 |
| rs2972144 | A | G | 0.3618 | -0.0911 | 0.0066 | 2.19E-43 | 1.62E-04 | 151.5148271 |
| rs11958808 | C | G | 0.3999 | 0.0403 | 0.0066 | 9.99E-10 | 1.62E-04 | 151.5148271 |
| rs12001437 | T | C | 0.6309 | -0.0402 | 0.0066 | 1.1E-09 | 1.62E-04 | 151.5148271 |
| rs2237895 | A | C | 0.573 | -0.0892 | 0.0066 | 1.15E-41 | 1.62E-04 | 151.5148271 |
| rs7240767 | T | C | 0.626 | -0.0372 | 0.0066 | 1.71E-08 | 1.62E-04 | 151.5148271 |
| rs4804833 | A | G | 0.3912 | 0.048 | 0.0066 | 3.42E-13 | 1.62E-04 | 151.5148271 |
| rs4686471 | T | C | 0.3887 | -0.0608 | 0.0066 | 3.05E-20 | 1.62E-04 | 151.5148271 |
| rs36138276 | A | G | 0.4992 | -0.0431 | 0.0066 | 6.4E-11 | 1.62E-04 | 151.5148271 |
| rs10882099 | T | C | 0.5879 | 0.1095 | 0.0065 | 7.73E-64 | 1.65E-04 | 153.8458244 |
| rs1127215 | T | C | 0.4153 | -0.0491 | 0.0065 | 3.92E-14 | 1.65E-04 | 153.8458244 |
| rs340874 | T | C | 0.449 | -0.0678 | 0.0065 | 1.56E-25 | 1.65E-04 | 153.8458244 |
| rs6545714 | A | G | 0.6081 | -0.0363 | 0.0065 | 2.25E-08 | 1.65E-04 | 153.8458244 |
| rs13389219 | T | C | 0.4014 | -0.0605 | 0.0065 | 1.17E-20 | 1.65E-04 | 153.8458244 |
| rs34341 | A | T | 0.4259 | -0.044 | 0.0065 | 1.22E-11 | 1.65E-04 | 153.8458244 |
| rs648795 | A | T | 0.4146 | 0.0391 | 0.0065 | 1.71E-09 | 1.65E-04 | 153.8458244 |
| rs1573090 | T | G | 0.5328 | 0.0458 | 0.0065 | 1.72E-12 | 1.65E-04 | 153.8458244 |
| rs34990153 | A | G | 0.5601 | 0.0476 | 0.0065 | 2.26E-13 | 1.65E-04 | 153.8458244 |
| rs2796441 | A | G | 0.406 | -0.0674 | 0.0065 | 2.97E-25 | 1.65E-04 | 153.8458244 |
| rs2812545 | A | G | 0.5206 | 0.0413 | 0.0065 | 1.99E-10 | 1.65E-04 | 153.8458244 |
| rs11856307 | A | C | 0.5706 | 0.047 | 0.0065 | 4.48E-13 | 1.65E-04 | 153.8458244 |
| rs12325539 | T | C | 0.5998 | -0.041 | 0.0065 | 2.69E-10 | 1.65E-04 | 153.8458244 |
| rs55872725 | T | C | 0.4175 | 0.122 | 0.0065 | 8.51E-79 | 1.65E-04 | 153.8458244 |
| rs13385171 | T | C | 0.4149 | 0.0357 | 0.0065 | 3.81E-08 | 1.65E-04 | 153.8458244 |
| rs329122 | A | G | 0.4292 | 0.0366 | 0.0065 | 1.72E-08 | 1.65E-04 | 153.8458244 |
| rs12719778 | T | C | 0.5365 | 0.0381 | 0.0065 | 4.38E-09 | 1.65E-04 | 153.8458244 |
| rs62563593 | A | G | 0.6063 | -0.0394 | 0.0065 | 1.28E-09 | 1.65E-04 | 153.8458244 |
| rs703972 | C | G | 0.4676 | -0.0698 | 0.0065 | 5.77E-27 | 1.65E-04 | 153.8458244 |
| rs10419627 | A | G | 0.591 | 0.0431 | 0.0065 | 3.15E-11 | 1.65E-04 | 153.8458244 |
| rs10406431 | A | G | 0.5626 | 0.0603 | 0.0065 | 1.56E-20 | 1.65E-04 | 153.8458244 |
| rs1999536 | C | G | 0.5732 | -0.0404 | 0.0065 | 4.86E-10 | 1.65E-04 | 153.8458244 |
| rs11699802 | T | C | 0.4649 | -0.0443 | 0.0065 | 8.84E-12 | 1.65E-04 | 153.8458244 |
| rs474513 | A | G | 0.5162 | 0.0399 | 0.0064 | 4.18E-10 | 1.67E-04 | 156.2496654 |
| rs1708302 | T | C | 0.4885 | -0.0909 | 0.0064 | 5.77E-46 | 1.67E-04 | 156.2496654 |
| rs10097617 | T | C | 0.485 | 0.0487 | 0.0064 | 2.44E-14 | 1.67E-04 | 156.2496654 |
| rs1381937 | A | C | 0.462 | 0.0442 | 0.0064 | 4.5E-12 | 1.67E-04 | 156.2496654 |
| rs7178762 | T | C | 0.5364 | -0.0387 | 0.0064 | 1.37E-09 | 1.67E-04 | 156.2496654 |
| rs4812034 | T | G | 0.5465 | 0.0416 | 0.0064 | 7.35E-11 | 1.67E-04 | 156.2496654 |
| rs243019 | T | C | 0.5359 | -0.0588 | 0.0064 | 3.37E-20 | 1.67E-04 | 156.2496654 |
| rs9873519 | T | C | 0.5384 | 0.0371 | 0.0064 | 6.29E-09 | 1.67E-04 | 156.2496654 |
| rs6821438 | A | G | 0.5342 | 0.0397 | 0.0064 | 5.1E-10 | 1.67E-04 | 156.2496654 |
| rs2215383 | T | C | 0.4625 | -0.0641 | 0.0064 | 1.06E-23 | 1.67E-04 | 156.2496654 |
| rs1705263 | A | C | 0.4367 | -0.0484 | 0.0064 | 3.51E-14 | 1.67E-04 | 156.2496654 |
| rs17522122 | T | G | 0.474 | 0.0356 | 0.0064 | 2.49E-08 | 1.67E-04 | 156.2496654 |
| rs2896177 | A | G | 0.5413 | -0.0367 | 0.0064 | 9.13E-09 | 1.67E-04 | 156.2496654 |
| rs13330951 | A | G | 0.5043 | 0.0361 | 0.0064 | 1.58E-08 | 1.67E-04 | 156.2496654 |
| **Glycated hemoglobin levels (HbA1c)** | | | | | | | | |
| rs655577 | T | C | 0.004 | -0.1094 | 0.0188 | 2.70E-09 | 3.62E-04 | 53.19076471 |
| rs12586299 | A | G | 0.009 | -0.1132 | 0.0148 | 7.31E-15 | 4.60E-04 | 67.56664707 |
| rs7947712 | A | G | 0.004 | -0.0638 | 0.0082 | 6.51E-17 | 8.30E-04 | 121.9495581 |
| rs117233107 | A | G | 0.02 | -0.047 | 0.0072 | 8.45E-11 | 9.45E-04 | 138.8869967 |
| rs35775737 | D | I | 0.028 | 0.0325 | 0.0052 | 5.23E-09 | 1.31E-03 | 192.3050724 |
| rs328185 | D | I | 0.032 | -0.0275 | 0.0048 | 2.62E-08 | 1.42E-03 | 208.3304951 |
| rs10169706 | T | C | 0.04 | 0.026 | 0.0046 | 1.48E-08 | 1.48E-03 | 217.3883427 |
| rs58415808 | T | C | 0.04 | 0.026 | 0.0046 | 1.48E-08 | 1.48E-03 | 217.3883427 |
| rs12134667 | T | C | 0.033 | -0.0273 | 0.0039 | 6.33E-12 | 1.74E-03 | 256.4067632 |
| rs13389076 | A | G | 0.034 | 0.0332 | 0.0038 | 3.04E-18 | 1.79E-03 | 263.1543096 |
| rs3827341 | T | G | 0.974 | 0.0239 | 0.0038 | 2.51E-10 | 1.79E-03 | 263.1543096 |
| rs6474359 | T | C | 0.978 | 0.0427 | 0.0038 | 1.91E-33 | 1.79E-03 | 263.1543096 |
| rs315800 | A | G | 0.945 | -0.0213 | 0.0037 | 1.65E-09 | 1.84E-03 | 270.2665883 |
| rs17531310 | A | G | 0.079 | -0.0208 | 0.0032 | 5.14E-11 | 2.12E-03 | 312.4957427 |
| rs138533393 | C | G | 0.049 | -0.0244 | 0.003 | 1.04E-16 | 2.27E-03 | 333.3287922 |
| rs61750929 | T | C | 0.041 | -0.0284 | 0.0029 | 9.49E-24 | 2.34E-03 | 344.8228885 |
| rs9444778 | T | G | 0.048 | -0.0272 | 0.0029 | 1.28E-21 | 2.34E-03 | 344.8228885 |
| rs9589030 | A | G | 0.959 | 0.0262 | 0.0029 | 8.66E-20 | 2.34E-03 | 344.8228885 |
| rs77410279 | T | C | 0.951 | 0.0241 | 0.0029 | 2.62E-16 | 2.34E-03 | 344.8228885 |
| rs148102365 | T | C | 0.914 | 0.0183 | 0.0028 | 1.44E-12 | 2.43E-03 | 357.1379916 |
| rs12825769 | A | T | 0.951 | 0.0242 | 0.0028 | 3.61E-19 | 2.43E-03 | 357.1379916 |
| rs9991640 | C | G | 0.045 | -0.0247 | 0.0028 | 1.12E-19 | 2.43E-03 | 357.1379916 |
| rs1800562 | A | G | 0.046 | -0.0383 | 0.0027 | 2.33E-50 | 2.52E-03 | 370.3653247 |
| rs7134216 | A | G | 0.059 | -0.0136 | 0.0026 | 2.08E-08 | 2.61E-03 | 384.6101448 |
| rs12332678 | D | I | 0.103 | -0.0134 | 0.0025 | 5.86E-09 | 2.72E-03 | 399.9945506 |
| rs76533333 | A | G | 0.913 | -0.0265 | 0.0025 | 2.81E-29 | 2.72E-03 | 399.9945506 |
| rs4706434 | A | G | 0.935 | 0.0287 | 0.0025 | 3.45E-35 | 2.72E-03 | 399.9945506 |
| rs17781758 | T | C | 0.074 | -0.0141 | 0.0024 | 3.86E-10 | 2.83E-03 | 416.6609902 |
| rs4727979 | A | C | 0.906 | 0.0121 | 0.0024 | 4.61E-08 | 2.83E-03 | 416.6609902 |
| rs73408859 | A | C | 0.906 | 0.0121 | 0.0024 | 4.61E-08 | 2.83E-03 | 416.6609902 |
| rs17340309 | T | C | 0.933 | -0.0129 | 0.0024 | 1.76E-08 | 2.83E-03 | 416.6609902 |
| rs481579 | A | G | 0.09 | -0.0125 | 0.0023 | 3.11E-09 | 2.95E-03 | 434.7766855 |
| rs35648279 | A | T | 0.912 | 0.0154 | 0.0023 | 9.44E-12 | 2.95E-03 | 434.7766855 |
| rs2256960 | C | G | 0.933 | 0.0146 | 0.0023 | 3.02E-11 | 2.95E-03 | 434.7766855 |
| rs9982693 | A | G | 0.925 | 0.0118 | 0.0023 | 4.70E-09 | 2.95E-03 | 434.7766855 |
| rs135324 | A | G | 0.918 | 0.0135 | 0.0023 | 2.61E-09 | 2.95E-03 | 434.7766855 |
| rs111855096 | T | C | 0.076 | -0.0131 | 0.0023 | 1.81E-08 | 2.95E-03 | 434.7766855 |
| rs12238969 | A | G | 0.91 | 0.0125 | 0.0023 | 3.38E-09 | 2.95E-03 | 434.7766855 |
| rs314318 | T | G | 0.09 | -0.0153 | 0.0023 | 1.28E-11 | 2.95E-03 | 434.7766855 |
| rs59235092 | T | C | 0.066 | 0.0129 | 0.0023 | 1.63E-08 | 2.95E-03 | 434.7766855 |
| rs78175314 | A | G | 0.076 | -0.0132 | 0.0023 | 1.41E-08 | 2.95E-03 | 434.7766855 |
| rs12945668 | T | G | 0.076 | -0.0132 | 0.0023 | 1.53E-08 | 2.95E-03 | 434.7766855 |
| rs2407283 | T | C | 0.079 | -0.0142 | 0.0022 | 1.18E-10 | 3.09E-03 | 454.5392621 |
| rs2748427 | A | G | 0.803 | -0.0307 | 0.0022 | 9.82E-49 | 3.09E-03 | 454.5392621 |
| rs6451723 | C | G | 0.917 | 0.0125 | 0.0021 | 1.94E-08 | 3.23E-03 | 476.1839888 |
| rs60811869 | D | I | 0.89 | 0.0171 | 0.0021 | 2.48E-20 | 3.23E-03 | 476.1839888 |
| rs16926246 | T | C | 0.136 | -0.0727 | 0.0021 | 1.00E-200 | 3.23E-03 | 476.1839888 |
| rs7547793 | A | C | 0.12 | -0.0118 | 0.0021 | 6.61E-09 | 3.23E-03 | 476.1839888 |
| rs10834282 | D | I | 0.834 | -0.0163 | 0.002 | 3.02E-18 | 3.39E-03 | 499.9931883 |
| rs4822454 | T | C | 0.156 | 0.0176 | 0.002 | 6.75E-22 | 3.39E-03 | 499.9931883 |
| rs1367173 | T | C | 0.106 | -0.0152 | 0.002 | 1.66E-14 | 3.39E-03 | 499.9931883 |
| rs2908277 | A | G | 0.117 | 0.0166 | 0.002 | 1.29E-18 | 3.39E-03 | 499.9931883 |
| rs13234131 | A | G | 0.876 | -0.0113 | 0.002 | 2.06E-09 | 3.39E-03 | 499.9931883 |
| rs80232704 | C | G | 0.786 | -0.0146 | 0.002 | 1.56E-12 | 3.39E-03 | 499.9931883 |
| rs55806536 | A | G | 0.858 | -0.0177 | 0.002 | 6.48E-22 | 3.39E-03 | 499.9931883 |
| rs115850317 | T | C | 0.141 | 0.0178 | 0.002 | 2.55E-22 | 3.39E-03 | 499.9931883 |
| rs16926828 | A | G | 0.148 | 0.0182 | 0.002 | 2.75E-23 | 3.39E-03 | 499.9931883 |
| rs926010 | A | C | 0.148 | 0.0183 | 0.002 | 1.69E-23 | 3.39E-03 | 499.9931883 |
| rs7068322 | A | T | 0.15 | 0.0183 | 0.002 | 1.86E-22 | 3.39E-03 | 499.9931883 |
| rs12129929 | T | G | 0.859 | -0.0182 | 0.002 | 6.75E-22 | 3.39E-03 | 499.9931883 |
| rs117912733 | T | G | 0.327 | 0.0184 | 0.002 | 3.37E-20 | 3.39E-03 | 499.9931883 |
| rs1964161 | A | G | 0.847 | -0.0182 | 0.002 | 4.48E-23 | 3.39E-03 | 499.9931883 |
| rs1932048 | A | G | 0.859 | 0.0634 | 0.002 | 1.00E-200 | 3.39E-03 | 499.9931883 |
| rs28812607 | A | G | 0.835 | -0.0148 | 0.002 | 3.59E-15 | 3.39E-03 | 499.9931883 |
| rs221696 | A | G | 0.165 | 0.0153 | 0.0019 | 8.38E-19 | 3.57E-03 | 526.3086193 |
| rs77936860 | A | C | 0.834 | -0.0153 | 0.0019 | 7.19E-19 | 3.57E-03 | 526.3086193 |
| rs72778336 | A | T | 0.876 | 0.0232 | 0.0019 | 8.68E-36 | 3.57E-03 | 526.3086193 |
| rs117935036 | A | G | 0.805 | -0.0097 | 0.0019 | 1.03E-09 | 3.57E-03 | 526.3086193 |
| rs77114091 | A | G | 0.894 | 0.0138 | 0.0019 | 4.58E-13 | 3.57E-03 | 526.3086193 |
| rs117190059 | D | I | 0.764 | -0.0119 | 0.0019 | 1.10E-10 | 3.57E-03 | 526.3086193 |
| rs13265258 | A | T | 0.807 | 0.0134 | 0.0019 | 4.59E-13 | 3.57E-03 | 526.3086193 |
| rs76223928 | C | G | 0.859 | 0.0093 | 0.0019 | 3.44E-08 | 3.57E-03 | 526.3086193 |
| rs17463581 | C | G | 0.858 | 0.0119 | 0.0019 | 4.40E-11 | 3.57E-03 | 526.3086193 |
| rs13170041 | A | G | 0.678 | -0.0179 | 0.0019 | 3.98E-22 | 3.57E-03 | 526.3086193 |
| rs11706712 | A | G | 0.794 | -0.0118 | 0.0018 | 4.87E-14 | 3.77E-03 | 555.547987 |
| rs4760682 | A | C | 0.817 | 0.0164 | 0.0018 | 3.20E-20 | 3.77E-03 | 555.547987 |
| rs13434761 | C | G | 0.869 | 0.011 | 0.0018 | 1.00E-09 | 3.77E-03 | 555.547987 |
| rs204995 | A | G | 0.781 | -0.0098 | 0.0018 | 1.93E-09 | 3.77E-03 | 555.547987 |
| rs143098299 | A | G | 0.187 | 0.0138 | 0.0018 | 2.64E-16 | 3.77E-03 | 555.547987 |
| rs10278474 | A | G | 0.194 | -0.0089 | 0.0017 | 1.69E-09 | 3.99E-03 | 588.2272803 |
| rs10811661 | T | C | 0.835 | 0.0128 | 0.0017 | 1.74E-14 | 3.99E-03 | 588.2272803 |
| rs149415187 | T | C | 0.223 | 0.0126 | 0.0017 | 8.27E-17 | 3.99E-03 | 588.2272803 |
| rs62028317 | T | C | 0.805 | 0.0117 | 0.0017 | 1.29E-13 | 3.99E-03 | 588.2272803 |
| rs12124079 | A | G | 0.179 | 0.0315 | 0.0017 | 3.50E-87 | 3.99E-03 | 588.2272803 |
| rs2971670 | T | C | 0.181 | 0.0316 | 0.0017 | 5.10E-88 | 3.99E-03 | 588.2272803 |
| rs79403657 | C | G | 0.823 | -0.009 | 0.0017 | 2.03E-08 | 3.99E-03 | 588.2272803 |
| rs968567 | T | C | 0.162 | -0.0108 | 0.0017 | 2.46E-09 | 3.99E-03 | 588.2272803 |
| rs1535464 | A | G | 0.212 | -0.0086 | 0.0017 | 1.11E-08 | 3.99E-03 | 588.2272803 |
| rs62523681 | A | T | 0.162 | -0.0165 | 0.0017 | 3.40E-20 | 3.99E-03 | 588.2272803 |
| rs13280469 | T | G | 0.678 | -0.0162 | 0.0017 | 5.26E-23 | 3.99E-03 | 588.2272803 |
| rs73522132 | A | G | 0.834 | 0.0111 | 0.0017 | 1.75E-10 | 3.99E-03 | 588.2272803 |
| rs9818758 | A | G | 0.204 | 0.0131 | 0.0017 | 1.49E-13 | 3.99E-03 | 588.2272803 |
| rs7608953 | T | C | 0.328 | 0.0149 | 0.0017 | 5.84E-21 | 3.99E-03 | 588.2272803 |
| rs10946402 | T | G | 0.831 | -0.0101 | 0.0016 | 1.12E-10 | 4.24E-03 | 624.9914854 |
| rs12053711 | A | G | 0.249 | 0.0238 | 0.0016 | 9.95E-54 | 4.24E-03 | 624.9914854 |
| rs78118741 | T | C | 0.207 | -0.008 | 0.0016 | 2.57E-08 | 4.24E-03 | 624.9914854 |
| rs3778321 | A | G | 0.176 | -0.0106 | 0.0016 | 4.18E-11 | 4.24E-03 | 624.9914854 |
| rs35928944 | A | G | 0.176 | -0.0106 | 0.0016 | 4.18E-11 | 4.24E-03 | 624.9914854 |
| rs7187287 | A | G | 0.176 | -0.0106 | 0.0016 | 4.18E-11 | 4.24E-03 | 624.9914854 |
| rs11257655 | T | C | 0.241 | 0.011 | 0.0016 | 1.91E-13 | 4.24E-03 | 624.9914854 |
| rs74764146 | D | I | 0.277 | -0.0085 | 0.0016 | 2.75E-09 | 4.24E-03 | 624.9914854 |
| rs11012032 | T | G | 0.832 | -0.0097 | 0.0016 | 1.69E-09 | 4.24E-03 | 624.9914854 |
| rs118140942 | T | C | 0.826 | 0.0118 | 0.0016 | 8.81E-16 | 4.24E-03 | 624.9914854 |
| rs67780013 | T | C | 0.761 | -0.0139 | 0.0016 | 1.51E-20 | 4.24E-03 | 624.9914854 |
| rs138467430 | A | G | 0.198 | 0.0121 | 0.0016 | 1.15E-12 | 4.24E-03 | 624.9914854 |
| rs17003548 | A | G | 0.194 | 0.0121 | 0.0016 | 1.77E-12 | 4.24E-03 | 624.9914854 |
| rs114828646 | T | C | 0.223 | 0.0125 | 0.0016 | 1.04E-15 | 4.24E-03 | 624.9914854 |
| rs59719825 | T | G | 0.277 | 0.0124 | 0.0016 | 2.34E-15 | 4.24E-03 | 624.9914854 |
| rs34287298 | T | C | 0.409 | -0.0169 | 0.0016 | 1.42E-25 | 4.24E-03 | 624.9914854 |
| rs10496954 | A | G | 0.309 | 0.015 | 0.0016 | 9.70E-24 | 4.24E-03 | 624.9914854 |
| rs267738 | T | G | 0.797 | 0.0109 | 0.0016 | 1.14E-11 | 4.24E-03 | 624.9914854 |
| rs35821610 | T | G | 0.19 | -0.0108 | 0.0016 | 2.05E-13 | 4.24E-03 | 624.9914854 |
| rs72740672 | A | G | 0.802 | 0.0125 | 0.0016 | 4.66E-17 | 4.24E-03 | 624.9914854 |
| rs115595512 | T | C | 0.253 | -0.0122 | 0.0016 | 4.57E-15 | 4.24E-03 | 624.9914854 |
| rs360147 | T | C | 0.264 | -0.0086 | 0.0015 | 2.08E-09 | 4.52E-03 | 666.6575844 |
| rs36043753 | T | C | 0.747 | 0.0084 | 0.0015 | 1.44E-08 | 4.52E-03 | 666.6575844 |
| rs9783598 | A | T | 0.332 | 0.0091 | 0.0015 | 4.83E-10 | 4.52E-03 | 666.6575844 |
| rs71436484 | C | G | 0.29 | -0.0113 | 0.0015 | 8.69E-13 | 4.52E-03 | 666.6575844 |
| rs831147 | A | C | 0.748 | -0.0087 | 0.0015 | 3.16E-10 | 4.52E-03 | 666.6575844 |
| rs651007 | T | C | 0.215 | 0.0108 | 0.0015 | 3.28E-15 | 4.52E-03 | 666.6575844 |
| rs1175549 | A | C | 0.786 | 0.0098 | 0.0015 | 7.13E-13 | 4.52E-03 | 666.6575844 |
| rs11643024 | A | G | 0.303 | 0.0084 | 0.0015 | 7.98E-10 | 4.52E-03 | 666.6575844 |
| rs10853129 | A | G | 0.678 | 0.0082 | 0.0015 | 4.05E-09 | 4.52E-03 | 666.6575844 |
| rs4499344 | A | G | 0.319 | -0.0111 | 0.0015 | 1.35E-12 | 4.52E-03 | 666.6575844 |
| rs6065561 | A | G | 0.281 | 0.0193 | 0.0015 | 2.37E-43 | 4.52E-03 | 666.6575844 |
| rs74322736 | A | G | 0.706 | -0.0193 | 0.0015 | 3.21E-43 | 4.52E-03 | 666.6575844 |
| rs4737009 | A | G | 0.262 | 0.0228 | 0.0015 | 8.29E-56 | 4.52E-03 | 666.6575844 |
| rs74045316 | T | C | 0.704 | -0.0168 | 0.0015 | 5.84E-32 | 4.52E-03 | 666.6575844 |
| rs61887899 | A | G | 0.7 | -0.0081 | 0.0015 | 1.54E-08 | 4.52E-03 | 666.6575844 |
| rs12667286 | D | I | 0.29 | 0.0277 | 0.0015 | 2.27E-75 | 4.52E-03 | 666.6575844 |
| rs34208293 | T | C | 0.35 | 0.0139 | 0.0015 | 2.79E-19 | 4.52E-03 | 666.6575844 |
| rs10830963 | C | G | 0.714 | -0.0197 | 0.0015 | 1.54E-36 | 4.52E-03 | 666.6575844 |
| rs1278769 | A | G | 0.231 | -0.0091 | 0.0015 | 5.52E-12 | 4.52E-03 | 666.6575844 |
| rs11719201 | T | C | 0.182 | -0.0129 | 0.0015 | 2.43E-18 | 4.52E-03 | 666.6575844 |
| rs3829109 | A | G | 0.276 | -0.0086 | 0.0015 | 2.68E-08 | 4.52E-03 | 666.6575844 |
| rs114291348 | A | T | 0.328 | 0.0142 | 0.0015 | 4.23E-23 | 4.52E-03 | 666.6575844 |
| rs9856473 | T | C | 0.672 | -0.0142 | 0.0015 | 1.56E-22 | 4.52E-03 | 666.6575844 |
| rs62495788 | C | G | 0.327 | 0.0141 | 0.0015 | 2.41E-22 | 4.52E-03 | 666.6575844 |
| rs6441849 | A | C | 0.798 | 0.0091 | 0.0015 | 1.69E-10 | 4.52E-03 | 666.6575844 |
| rs459369 | A | G | 0.784 | -0.0074 | 0.0015 | 1.27E-08 | 4.52E-03 | 666.6575844 |
| rs1850734 | T | C | 0.591 | -0.0071 | 0.0014 | 1.28E-09 | 4.84E-03 | 714.2759833 |
| rs140582954 | C | G | 0.425 | 0.0084 | 0.0014 | 2.02E-10 | 4.84E-03 | 714.2759833 |
| rs17533945 | T | C | 0.582 | -0.0128 | 0.0014 | 1.62E-23 | 4.84E-03 | 714.2759833 |
| rs11096873 | T | C | 0.503 | 0.0092 | 0.0014 | 2.72E-11 | 4.84E-03 | 714.2759833 |
| rs72751832 | T | C | 0.587 | -0.0076 | 0.0014 | 1.99E-08 | 4.84E-03 | 714.2759833 |
| rs72639766 | T | C | 0.587 | -0.0076 | 0.0014 | 1.99E-08 | 4.84E-03 | 714.2759833 |
| rs7903146 | T | C | 0.307 | 0.0133 | 0.0014 | 1.04E-22 | 4.84E-03 | 714.2759833 |
| rs7696107 | T | C | 0.764 | 0.0126 | 0.0014 | 5.64E-17 | 4.84E-03 | 714.2759833 |
| rs6877043 | T | C | 0.638 | 0.0085 | 0.0014 | 1.99E-10 | 4.84E-03 | 714.2759833 |
| rs13089972 | A | T | 0.584 | 0.0111 | 0.0014 | 1.87E-15 | 4.84E-03 | 714.2759833 |
| rs11248914 | T | C | 0.698 | 0.0114 | 0.0014 | 1.42E-14 | 4.84E-03 | 714.2759833 |
| rs72875779 | T | C | 0.569 | 0.0144 | 0.0014 | 5.87E-31 | 4.84E-03 | 714.2759833 |
| rs73228737 | A | G | 0.39 | -0.0102 | 0.0014 | 2.98E-13 | 4.84E-03 | 714.2759833 |
| rs62144745 | T | C | 0.584 | -0.011 | 0.0014 | 2.68E-18 | 4.84E-03 | 714.2759833 |
| rs8129689 | T | C | 0.584 | -0.011 | 0.0014 | 2.68E-18 | 4.84E-03 | 714.2759833 |
| rs1508527 | C | G | 0.434 | 0.011 | 0.0014 | 1.70E-17 | 4.84E-03 | 714.2759833 |
| rs658303 | A | G | 0.443 | 0.011 | 0.0014 | 1.39E-17 | 4.84E-03 | 714.2759833 |
| rs2120314 | A | G | 0.566 | -0.0111 | 0.0014 | 1.13E-17 | 4.84E-03 | 714.2759833 |
| rs10909012 | T | G | 0.409 | 0.0111 | 0.0014 | 7.62E-19 | 4.84E-03 | 714.2759833 |
| rs13228313 | T | C | 0.414 | 0.011 | 0.0014 | 1.06E-17 | 4.84E-03 | 714.2759833 |
| rs11039165 | A | G | 0.73 | 0.0083 | 0.0014 | 8.16E-09 | 4.84E-03 | 714.2759833 |
| rs1966197 | T | C | 0.371 | 0.0075 | 0.0014 | 1.73E-08 | 4.84E-03 | 714.2759833 |
| rs12487379 | A | G | 0.405 | 0.0078 | 0.0014 | 2.18E-08 | 4.84E-03 | 714.2759833 |
| rs113717347 | A | G | 0.612 | -0.0074 | 0.0014 | 3.46E-08 | 4.84E-03 | 714.2759833 |
| rs150984598 | C | G | 0.39 | -0.0083 | 0.0014 | 4.16E-09 | 4.84E-03 | 714.2759833 |
| rs1850560 | A | G | 0.632 | -0.0086 | 0.0014 | 1.17E-10 | 4.84E-03 | 714.2759833 |
| rs6472158 | A | G | 0.614 | -0.0088 | 0.0014 | 2.06E-11 | 4.84E-03 | 714.2759833 |
| rs452306 | T | C | 0.627 | -0.0098 | 0.0014 | 5.51E-13 | 4.84E-03 | 714.2759833 |
| rs7198799 | T | C | 0.281 | 0.0083 | 0.0014 | 4.76E-09 | 4.84E-03 | 714.2759833 |
| rs112944361 | A | G | 0.39 | 0.0079 | 0.0014 | 2.50E-08 | 4.84E-03 | 714.2759833 |
| rs9909940 | T | C | 0.323 | 0.0322 | 0.0014 | 1.43E-116 | 4.84E-03 | 714.2759833 |
| rs73145172 | D | I | 0.52 | 0.0126 | 0.0014 | 9.23E-19 | 4.84E-03 | 714.2759833 |
| rs34369987 | T | G | 0.661 | 0.0157 | 0.0014 | 2.57E-30 | 4.84E-03 | 714.2759833 |
| rs118059355 | A | G | 0.197 | -0.012 | 0.0014 | 1.20E-16 | 4.84E-03 | 714.2759833 |
| rs13134327 | A | G | 0.331 | 0.0144 | 0.0014 | 2.81E-26 | 4.84E-03 | 714.2759833 |
| rs857725 | T | G | 0.723 | -0.0208 | 0.0014 | 5.43E-55 | 4.84E-03 | 714.2759833 |
| rs560887 | T | C | 0.306 | -0.0307 | 0.0014 | 5.55E-122 | 4.84E-03 | 714.2759833 |
| rs6804915 | A | C | 0.288 | -0.0108 | 0.0014 | 2.76E-16 | 4.84E-03 | 714.2759833 |
| rs4980325 | T | G | 0.532 | 0.0108 | 0.0014 | 4.70E-14 | 4.84E-03 | 714.2759833 |
| rs78058400 | C | G | 0.328 | 0.0083 | 0.0014 | 6.97E-10 | 4.84E-03 | 714.2759833 |
| rs2338756 | T | G | 0.46 | 0.0121 | 0.0014 | 3.29E-23 | 4.84E-03 | 714.2759833 |
| rs873290 | T | C | 0.416 | 0.011 | 0.0014 | 5.81E-18 | 4.84E-03 | 714.2759833 |
| rs243123 | A | C | 0.668 | 0.0081 | 0.0014 | 4.13E-09 | 4.84E-03 | 714.2759833 |
| rs4548859 | A | G | 0.379 | 0.0077 | 0.0014 | 2.50E-08 | 4.84E-03 | 714.2759833 |
| rs144926897 | T | C | 0.606 | 0.0088 | 0.0014 | 2.77E-10 | 4.84E-03 | 714.2759833 |
| rs4902387 | A | G | 0.619 | -0.009 | 0.0014 | 1.08E-11 | 4.84E-03 | 714.2759833 |
| rs7539528 | T | C | 0.381 | 0.0091 | 0.0014 | 9.13E-12 | 4.84E-03 | 714.2759833 |
| rs78139179 | T | C | 0.379 | 0.0092 | 0.0014 | 6.58E-12 | 4.84E-03 | 714.2759833 |
| rs2102339 | T | C | 0.334 | -0.0087 | 0.0014 | 3.42E-10 | 4.84E-03 | 714.2759833 |
| rs4272839 | A | G | 0.734 | -0.0273 | 0.0014 | 3.17E-80 | 4.84E-03 | 714.2759833 |
| rs1990812 | D | I | 0.451 | -0.0116 | 0.0014 | 1.35E-16 | 4.84E-03 | 714.2759833 |
| rs867378 | A | G | 0.441 | 0.0162 | 0.0014 | 7.72E-31 | 4.84E-03 | 714.2759833 |
| rs11558471 | A | G | 0.707 | 0.0151 | 0.0014 | 3.38E-25 | 4.84E-03 | 714.2759833 |
| rs9376090 | T | C | 0.728 | 0.0247 | 0.0014 | 1.90E-62 | 4.84E-03 | 714.2759833 |
| rs2130671 | D | I | 0.443 | 0.0127 | 0.0014 | 3.24E-22 | 4.84E-03 | 714.2759833 |
| rs10231021 | A | T | 0.492 | 0.0089 | 0.0013 | 8.69E-14 | 5.21E-03 | 769.2202897 |
| rs62356127 | T | C | 0.484 | -0.0082 | 0.0013 | 2.72E-12 | 5.21E-03 | 769.2202897 |
| rs72935122 | T | C | 0.408 | -0.0075 | 0.0013 | 4.92E-09 | 5.21E-03 | 769.2202897 |
| rs34184773 | T | G | 0.537 | 0.0104 | 0.0013 | 4.95E-13 | 5.21E-03 | 769.2202897 |
| rs142998447 | T | C | 0.443 | -0.0088 | 0.0013 | 4.40E-13 | 5.21E-03 | 769.2202897 |
| rs150642717 | T | C | 0.522 | -0.0084 | 0.0013 | 1.29E-12 | 5.21E-03 | 769.2202897 |
| rs8110303 | T | C | 0.518 | 0.0083 | 0.0013 | 2.26E-12 | 5.21E-03 | 769.2202897 |
| rs7437341 | A | G | 0.57 | 0.0106 | 0.0013 | 7.75E-19 | 5.21E-03 | 769.2202897 |
| rs2829235 | A | G | 0.66 | -0.0083 | 0.0013 | 4.40E-10 | 5.21E-03 | 769.2202897 |
| rs76519057 | A | G | 0.61 | 0.0134 | 0.0013 | 2.41E-28 | 5.21E-03 | 769.2202897 |
| rs235960 | A | G | 0.327 | 0.0078 | 0.0013 | 1.25E-09 | 5.21E-03 | 769.2202897 |
| rs855791 | A | G | 0.4 | 0.0188 | 0.0013 | 1.34E-56 | 5.21E-03 | 769.2202897 |
| rs76559790 | T | C | 0.534 | 0.012 | 0.0013 | 8.12E-21 | 5.21E-03 | 769.2202897 |
| rs1335896 | T | G | 0.52 | 0.022 | 0.0013 | 4.25E-71 | 5.21E-03 | 769.2202897 |
| rs537719859 | A | C | 0.4 | -0.0118 | 0.0013 | 7.71E-19 | 5.21E-03 | 769.2202897 |
| rs737092 | T | C | 0.501 | -0.0073 | 0.0013 | 7.57E-09 | 5.21E-03 | 769.2202897 |
| rs7114296 | A | G | 0.566 | -0.0101 | 0.0013 | 5.19E-17 | 5.21E-03 | 769.2202897 |
| rs12614770 | T | G | 0.488 | 0.0281 | 0.0013 | 6.36E-105 | 5.21E-03 | 769.2202897 |
| rs74027980 | T | C | 0.578 | 0.019 | 0.0013 | 1.21E-50 | 5.21E-03 | 769.2202897 |
| rs2289356 | T | C | 0.413 | -0.0194 | 0.0013 | 1.71E-53 | 5.21E-03 | 769.2202897 |
| rs59519532 | A | G | 0.402 | 0.0163 | 0.0013 | 8.39E-40 | 5.21E-03 | 769.2202897 |
| rs837763 | T | C | 0.578 | 0.0176 | 0.0013 | 5.20E-38 | 5.21E-03 | 769.2202897 |
| rs11598058 | A | G | 0.536 | 0.0109 | 0.0013 | 6.19E-18 | 5.21E-03 | 769.2202897 |
| rs7042939 | A | G | 0.418 | 0.0102 | 0.0013 | 1.50E-15 | 5.21E-03 | 769.2202897 |
| rs138515317 | T | C | 0.524 | -0.0087 | 0.0013 | 8.84E-13 | 5.21E-03 | 769.2202897 |
| rs608793 | T | C | 0.479 | 0.0065 | 0.0013 | 4.55E-08 | 5.21E-03 | 769.2202897 |
| rs162481 | T | C | 0.563 | 0.0085 | 0.0013 | 2.76E-12 | 5.21E-03 | 769.2202897 |
| rs200548 | C | G | 0.55 | 0.0089 | 0.0013 | 4.80E-14 | 5.21E-03 | 769.2202897 |
| rs2001846 | T | C | 0.471 | -0.0069 | 0.0013 | 8.58E-10 | 5.21E-03 | 769.2202897 |
| rs248160 | C | G | 0.504 | 0.0077 | 0.0013 | 2.29E-08 | 5.21E-03 | 769.2202897 |
| rs13386851 | A | G | 0.592 | 0.0088 | 0.0013 | 2.83E-12 | 5.21E-03 | 769.2202897 |
| rs13392177 | T | C | 0.475 | -0.0071 | 0.0013 | 1.63E-08 | 5.21E-03 | 769.2202897 |
| rs2353605 | A | G | 0.462 | -0.0106 | 0.0013 | 2.42E-13 | 5.21E-03 | 769.2202897 |
| rs12491937 | A | G | 0.555 | 0.009 | 0.0013 | 1.42E-13 | 5.21E-03 | 769.2202897 |
| rs7630803 | C | G | 0.39 | -0.0133 | 0.0013 | 7.41E-28 | 5.21E-03 | 769.2202897 |
| rs4800881 | C | G | 0.421 | -0.012 | 0.0013 | 1.14E-21 | 5.21E-03 | 769.2202897 |
| rs10185886 | A | G | 0.61 | 0.0133 | 0.0013 | 3.19E-28 | 5.21E-03 | 769.2202897 |
| rs11048350 | A | G | 0.61 | 0.0133 | 0.0013 | 3.19E-28 | 5.21E-03 | 769.2202897 |
| rs117966122 | C | G | 0.389 | -0.0134 | 0.0013 | 1.37E-28 | 5.21E-03 | 769.2202897 |
| rs10491883 | A | G | 0.67 | -0.0075 | 0.0013 | 2.28E-09 | 5.21E-03 | 769.2202897 |
| rs10784182 | A | C | 0.368 | -0.0084 | 0.0013 | 2.67E-09 | 5.21E-03 | 769.2202897 |
| rs11127707 | T | C | 0.372 | -0.0257 | 0.0013 | 1.18E-82 | 5.21E-03 | 769.2202897 |
| rs1781759 | A | G | 0.584 | -0.0169 | 0.0013 | 1.06E-42 | 5.21E-03 | 769.2202897 |
| rs348033 | A | C | 0.584 | -0.0169 | 0.0013 | 5.15E-43 | 5.21E-03 | 769.2202897 |
| rs7127313 | T | C | 0.336 | 0.0066 | 0.0013 | 4.85E-08 | 5.21E-03 | 769.2202897 |
| rs6766187 | T | C | 0.534 | 0.0087 | 0.0013 | 1.11E-12 | 5.21E-03 | 769.2202897 |
| rs6967235 | T | C | 0.512 | -0.0114 | 0.0013 | 2.24E-20 | 5.21E-03 | 769.2202897 |
| rs7429205 | T | G | 0.512 | -0.0115 | 0.0013 | 1.79E-20 | 5.21E-03 | 769.2202897 |
| rs74931366 | T | C | 0.483 | -0.0191 | 0.0013 | 1.31E-56 | 5.21E-03 | 769.2202897 |
| rs76216148 | T | C | 0.511 | 0.0187 | 0.0013 | 1.35E-53 | 5.21E-03 | 769.2202897 |
| rs340882 | C | G | 0.42 | -0.0084 | 0.0013 | 1.48E-10 | 5.21E-03 | 769.2202897 |
| rs117600549 | T | C | 0.475 | 0.0087 | 0.0013 | 7.31E-13 | 5.21E-03 | 769.2202897 |
| rs56016156 | T | C | 0.64 | 0.0271 | 0.0013 | 2.32E-102 | 5.21E-03 | 769.2202897 |
| rs4906745 | A | G | 0.483 | -0.0081 | 0.0012 | 1.68E-11 | 5.64E-03 | 833.3219805 |
| **Fasting glucose (FG)** | | | | | | | | |
| rs79896666 | A | C | 0.941 | 0.0283 | 0.0039 | 3.67E-13 | 1.28E-03 | 256.4077003 |
| rs17044206 | D | I | 0.922 | 0.0305 | 0.0031 | 3.32E-25 | 1.61E-03 | 322.5774294 |
| rs12784552 | A | G | 0.924 | 0.0329 | 0.003 | 2.86E-31 | 1.66E-03 | 333.3300103 |
| rs80232704 | C | G | 0.786 | -0.0508 | 0.0025 | 5.50E-91 | 1.99E-03 | 399.9960124 |
| rs11819063 | T | G | 0.839 | 0.0148 | 0.0024 | 1.45E-10 | 2.07E-03 | 416.6625129 |
| rs2232647 | A | T | 0.836 | 0.0141 | 0.0024 | 4.02E-10 | 2.07E-03 | 416.6625129 |
| rs564046898 | A | G | 0.145 | 0.0141 | 0.0023 | 3.27E-09 | 2.16E-03 | 434.7782743 |
| rs113129541 | T | G | 0.854 | -0.0138 | 0.0023 | 1.04E-08 | 2.16E-03 | 434.7782743 |
| rs192743856 | T | C | 0.131 | 0.0135 | 0.0023 | 2.48E-08 | 2.16E-03 | 434.7782743 |
| rs59105820 | D | I | 0.235 | 0.0181 | 0.0023 | 1.71E-16 | 2.16E-03 | 434.7782743 |
| rs1060043 | T | C | 0.854 | -0.0136 | 0.0023 | 5.48E-09 | 2.16E-03 | 434.7782743 |
| rs7153090 | T | C | 0.846 | -0.0148 | 0.0023 | 4.40E-10 | 2.16E-03 | 434.7782743 |
| rs9783897 | A | T | 0.846 | 0.0183 | 0.0023 | 5.25E-13 | 2.16E-03 | 434.7782743 |
| rs35777815 | T | C | 0.146 | -0.018 | 0.0023 | 3.24E-12 | 2.16E-03 | 434.7782743 |
| rs11603349 | T | C | 0.832 | 0.0236 | 0.0022 | 3.12E-25 | 2.26E-03 | 454.5409232 |
| rs527644 | T | C | 0.245 | -0.0122 | 0.0022 | 3.09E-08 | 2.26E-03 | 454.5409232 |
| rs8074028 | A | T | 0.234 | 0.0176 | 0.0022 | 3.55E-16 | 2.26E-03 | 454.5409232 |
| rs112053160 | A | G | 0.234 | 0.0176 | 0.0022 | 1.75E-17 | 2.26E-03 | 454.5409232 |
| rs34762197 | A | C | 0.852 | -0.0138 | 0.0022 | 4.26E-09 | 2.26E-03 | 454.5409232 |
| rs10877579 | A | G | 0.227 | -0.015 | 0.0022 | 5.63E-11 | 2.26E-03 | 454.5409232 |
| rs3842753 | T | G | 0.28 | 0.0134 | 0.0022 | 2.84E-09 | 2.26E-03 | 454.5409232 |
| rs34302671 | T | C | 0.28 | -0.0132 | 0.0022 | 3.76E-09 | 2.26E-03 | 454.5409232 |
| rs7193602 | T | G | 0.212 | 0.0119 | 0.0021 | 3.15E-08 | 2.37E-03 | 476.185729 |
| rs7302925 | A | G | 0.231 | 0.0111 | 0.0021 | 2.00E-08 | 2.37E-03 | 476.185729 |
| rs17270243 | A | G | 0.76 | -0.0104 | 0.0021 | 3.62E-08 | 2.37E-03 | 476.185729 |
| rs180893004 | T | C | 0.759 | 0.0112 | 0.0021 | 2.08E-08 | 2.37E-03 | 476.185729 |
| rs149557155 | T | C | 0.734 | -0.011 | 0.0021 | 2.00E-08 | 2.37E-03 | 476.185729 |
| rs546243106 | T | C | 0.255 | 0.013 | 0.0021 | 5.05E-09 | 2.37E-03 | 476.185729 |
| rs144013274 | A | G | 0.8 | 0.0189 | 0.0021 | 3.09E-18 | 2.37E-03 | 476.185729 |
| rs116940860 | D | I | 0.351 | -0.018 | 0.002 | 1.05E-19 | 2.49E-03 | 499.9950155 |
| rs11619319 | A | G | 0.766 | -0.0173 | 0.002 | 3.41E-20 | 2.49E-03 | 499.9950155 |
| rs3783347 | T | G | 0.189 | -0.0136 | 0.002 | 1.39E-11 | 2.49E-03 | 499.9950155 |
| rs71432933 | D | I | 0.273 | 0.0189 | 0.002 | 1.04E-20 | 2.49E-03 | 499.9950155 |
| rs2238435 | C | G | 0.381 | 0.0112 | 0.0019 | 3.82E-09 | 2.62E-03 | 526.3105426 |
| rs238415 | T | C | 0.212 | 0.0162 | 0.0019 | 1.99E-15 | 2.62E-03 | 526.3105426 |
| rs62007299 | A | G | 0.677 | 0.013 | 0.0019 | 1.73E-09 | 2.62E-03 | 526.3105426 |
| rs76215346 | T | C | 0.708 | 0.0124 | 0.0019 | 1.03E-10 | 2.62E-03 | 526.3105426 |
| rs7903146 | T | C | 0.307 | 0.0259 | 0.0019 | 2.00E-35 | 2.62E-03 | 526.3105426 |
| rs7909740 | A | C | 0.216 | 0.0166 | 0.0019 | 2.37E-16 | 2.62E-03 | 526.3105426 |
| rs58909947 | T | C | 0.748 | 0.0149 | 0.0019 | 3.72E-15 | 2.62E-03 | 526.3105426 |
| rs115652575 | A | G | 0.27 | -0.0109 | 0.0019 | 2.78E-09 | 2.62E-03 | 526.3105426 |
| rs73145097 | T | C | 0.319 | -0.0126 | 0.0019 | 5.23E-09 | 2.62E-03 | 526.3105426 |
| rs8021593 | A | G | 0.621 | -0.0112 | 0.0019 | 1.37E-08 | 2.62E-03 | 526.3105426 |
| rs34208293 | T | C | 0.35 | 0.0498 | 0.0019 | 1.99E-140 | 2.62E-03 | 526.3105426 |
| rs11020124 | T | C | 0.722 | -0.0599 | 0.0019 | 1.00E-200 | 2.62E-03 | 526.3105426 |
| rs6477497 | A | G | 0.38 | 0.0104 | 0.0019 | 3.27E-09 | 2.62E-03 | 526.3105426 |
| rs11610045 | A | G | 0.454 | 0.0144 | 0.0019 | 3.26E-13 | 2.62E-03 | 526.3105426 |
| rs3766198 | T | C | 0.695 | -0.0172 | 0.0018 | 2.44E-22 | 2.76E-03 | 555.5500172 |
| rs141518854 | A | G | 0.695 | -0.0173 | 0.0018 | 1.06E-22 | 2.76E-03 | 555.5500172 |
| rs10838693 | C | G | 0.314 | 0.0177 | 0.0018 | 3.44E-23 | 2.76E-03 | 555.5500172 |
| rs66608047 | T | C | 0.298 | 0.0122 | 0.0018 | 1.73E-10 | 2.76E-03 | 555.5500172 |
| rs13103660 | T | C | 0.695 | -0.0124 | 0.0018 | 4.04E-11 | 2.76E-03 | 555.5500172 |
| rs77467424 | T | G | 0.723 | 0.0113 | 0.0018 | 3.53E-10 | 2.76E-03 | 555.5500172 |
| rs144926897 | T | C | 0.606 | 0.0163 | 0.0018 | 5.36E-19 | 2.76E-03 | 555.5500172 |
| rs11020230 | A | G | 0.438 | -0.01 | 0.0018 | 1.06E-08 | 2.76E-03 | 555.5500172 |
| rs6489811 | A | G | 0.488 | -0.011 | 0.0018 | 3.27E-09 | 2.76E-03 | 555.5500172 |
| rs150984598 | C | G | 0.39 | -0.0161 | 0.0017 | 1.93E-19 | 2.92E-03 | 588.22943 |
| rs310612 | T | C | 0.431 | 0.0101 | 0.0017 | 1.30E-09 | 2.92E-03 | 588.22943 |
| rs1217636 | A | C | 0.352 | -0.0138 | 0.0017 | 4.74E-15 | 2.92E-03 | 588.22943 |
| rs3933514 | T | C | 0.555 | -0.0142 | 0.0017 | 1.15E-15 | 2.92E-03 | 588.22943 |
| rs2762488 | A | G | 0.468 | 0.0154 | 0.0017 | 5.76E-18 | 2.92E-03 | 588.22943 |
| rs12310116 | A | G | 0.482 | 0.0243 | 0.0017 | 2.83E-39 | 2.92E-03 | 588.22943 |
| rs1859437 | T | C | 0.608 | 0.0153 | 0.0017 | 1.86E-19 | 2.92E-03 | 588.22943 |
| rs174583 | T | C | 0.375 | -0.0168 | 0.0017 | 3.37E-22 | 2.92E-03 | 588.22943 |
| rs12898997 | T | C | 0.598 | -0.0098 | 0.0017 | 4.64E-09 | 2.92E-03 | 588.22943 |
| rs78150385 | A | G | 0.468 | -0.0127 | 0.0017 | 5.44E-13 | 2.92E-03 | 588.22943 |
| rs8090349 | A | G | 0.493 | -0.0228 | 0.0016 | 5.74E-38 | 3.11E-03 | 624.9937694 |
| rs60804270 | C | G | 0.431 | -0.0215 | 0.0016 | 1.19E-35 | 3.11E-03 | 624.9937694 |
| rs11598058 | A | G | 0.536 | 0.0391 | 0.0016 | 2.13E-115 | 3.11E-03 | 624.9937694 |
| rs10838524 | A | G | 0.48 | 0.0238 | 0.0016 | 1.56E-40 | 3.11E-03 | 624.9937694 |
| rs7163757 | T | C | 0.433 | -0.0217 | 0.0016 | 2.64E-36 | 3.11E-03 | 624.9937694 |
| rs1595979 | A | G | 0.57 | 0.0215 | 0.0016 | 1.73E-35 | 3.11E-03 | 624.9937694 |
| rs138515317 | T | C | 0.524 | -0.0143 | 0.0016 | 4.32E-16 | 3.11E-03 | 624.9937694 |
| **Two-hour glucose (2hGlu)** | | | | | | | | |
| rs117643180 | A | C | 0.033 | 0.234 | 0.0327 | 7.31E-14 | 4.82E-04 | 30.58007499 |
| rs71327027 | D | I | 0.096 | 0.0893 | 0.0134 | 5.24E-12 | 1.18E-03 | 74.62451136 |
| rs10517152 | A | G | 0.888 | -0.0897 | 0.0131 | 1.52E-12 | 1.20E-03 | 76.33346964 |
| rs6039413 | A | G | 0.075 | -0.0707 | 0.0126 | 3.13E-09 | 1.25E-03 | 79.36257558 |
| rs6972708 | A | G | 0.083 | -0.0682 | 0.0124 | 6.06E-09 | 1.27E-03 | 80.64261712 |
| rs2126259 | T | C | 0.091 | -0.0716 | 0.0121 | 2.98E-10 | 1.30E-03 | 82.64202085 |
| rs11881679 | A | T | 0.104 | -0.0655 | 0.0119 | 5.88E-09 | 1.32E-03 | 84.03096238 |
| rs12124079 | A | G | 0.179 | 0.1037 | 0.0105 | 2.26E-24 | 1.50E-03 | 95.23509069 |
| rs1678722 | A | G | 0.718 | 0.0576 | 0.0102 | 3.41E-08 | 1.54E-03 | 98.03612277 |
| rs151033601 | A | G | 0.71 | 0.0564 | 0.01 | 2.35E-08 | 1.57E-03 | 99.99684523 |
| rs12053711 | A | G | 0.249 | 0.0743 | 0.0099 | 2.17E-14 | 1.59E-03 | 101.0069144 |
| rs1800437 | C | G | 0.208 | 0.1004 | 0.0099 | 4.79E-26 | 1.59E-03 | 101.0069144 |
| rs878521 | A | G | 0.249 | 0.099 | 0.0094 | 1.25E-28 | 1.68E-03 | 106.3796226 |
| rs11708067 | A | G | 0.823 | 0.0872 | 0.0093 | 1.98E-22 | 1.69E-03 | 107.5234895 |
| rs12000334 | D | I | 0.259 | 0.0449 | 0.009 | 3.80E-08 | 1.75E-03 | 111.1076058 |
| rs299702 | T | C | 0.284 | 0.0537 | 0.009 | 1.80E-09 | 1.75E-03 | 111.1076058 |
| rs118059355 | A | G | 0.197 | -0.0737 | 0.009 | 1.67E-17 | 1.75E-03 | 111.1076058 |
| rs12692738 | T | C | 0.735 | 0.0486 | 0.009 | 2.72E-08 | 1.75E-03 | 111.1076058 |
| rs73123986 | A | G | 0.724 | -0.0506 | 0.0088 | 3.81E-09 | 1.79E-03 | 113.6327787 |
| rs7903146 | T | C | 0.307 | 0.0854 | 0.0087 | 2.79E-26 | 1.81E-03 | 114.9389026 |
| rs7696107 | T | C | 0.764 | 0.0676 | 0.0087 | 5.36E-15 | 1.81E-03 | 114.9389026 |
| rs550057 | T | C | 0.286 | 0.0526 | 0.0085 | 3.62E-11 | 1.85E-03 | 117.6433473 |
| rs76559790 | T | C | 0.534 | 0.053 | 0.0083 | 9.59E-12 | 1.90E-03 | 120.4781268 |
| rs35738193 | A | T | 0.289 | 0.0552 | 0.0082 | 2.71E-11 | 1.92E-03 | 121.9473722 |
| rs2649999 | T | C | 0.359 | 0.0498 | 0.0082 | 2.01E-10 | 1.92E-03 | 121.9473722 |
| rs9808924 | A | G | 0.286 | 0.0559 | 0.0082 | 1.56E-11 | 1.92E-03 | 121.9473722 |
| rs77866501 | A | G | 0.566 | -0.0426 | 0.0081 | 7.90E-09 | 1.94E-03 | 123.4528953 |
| rs1335896 | T | G | 0.52 | 0.0754 | 0.008 | 5.94E-23 | 1.97E-03 | 124.9960565 |
| rs114023299 | T | C | 0.48 | 0.0506 | 0.008 | 2.24E-10 | 1.97E-03 | 124.9960565 |
| rs10784277 | T | C | 0.48 | 0.0506 | 0.008 | 2.24E-10 | 1.97E-03 | 124.9960565 |
| rs117767193 | A | C | 0.528 | -0.0482 | 0.0079 | 8.61E-10 | 1.99E-03 | 126.5782851 |
| rs4148646 | C | G | 0.336 | 0.0397 | 0.0078 | 4.39E-08 | 2.02E-03 | 128.2010836 |
| rs162481 | T | C | 0.563 | 0.0428 | 0.0078 | 6.91E-09 | 2.02E-03 | 128.2010836 |
| rs11647977 | A | G | 0.573 | -0.046 | 0.0077 | 1.97E-09 | 2.04E-03 | 129.8660328 |
| rs80233087 | T | C | 0.552 | -0.0532 | 0.0077 | 1.10E-11 | 2.04E-03 | 129.8660328 |
| rs17271305 | A | G | 0.591 | -0.0587 | 0.0077 | 2.88E-14 | 2.04E-03 | 129.8660328 |
| rs4388340 | A | G | 0.545 | -0.0472 | 0.0076 | 1.77E-09 | 2.07E-03 | 131.5747964 |
| rs117600549 | T | C | 0.475 | 0.0408 | 0.0076 | 3.61E-08 | 2.07E-03 | 131.5747964 |
| rs7097350 | T | C | 0.536 | -0.0493 | 0.0076 | 1.91E-10 | 2.07E-03 | 131.5747964 |
| rs138515317 | T | C | 0.524 | -0.0407 | 0.0076 | 3.27E-08 | 2.07E-03 | 131.5747964 |
| **Insulin fold change during an oral glucose tolerance test (adjusted for BMI) (IFC)** | | | | | | | | |
| rs117643180 | A | C | 0.0231 | 0.1428 | 0.0221 | 1.11E-10 | 8.48E-04 | 45.24717047 |
| rs6039413 | A | G | 0.1161 | -0.0691 | 0.0103 | 1.61E-11 | 1.82E-03 | 97.0837347 |
| rs6972708 | A | G | 0.1212 | -0.0677 | 0.0101 | 1.65E-11 | 1.85E-03 | 99.00618489 |
| rs1387153 | T | C | 0.3023 | 0.0438 | 0.0069 | 2.25E-10 | 2.71E-03 | 144.9220967 |
| rs7012814 | A | G | 0.4796 | 0.0481 | 0.0065 | 1.35E-13 | 2.88E-03 | 153.8403796 |
| rs60804270 | C | G | 0.4603 | -0.042 | 0.0064 | 5.20E-11 | 2.92E-03 | 156.2441355 |
| rs7167878 | A | C | 0.4606 | -0.0426 | 0.0064 | 2.76E-11 | 2.92E-03 | 156.2441355 |
| rs1595979 | A | G | 0.5392 | 0.0426 | 0.0064 | 2.76E-11 | 2.92E-03 | 156.2441355 |
| **Modified Stumvoll Insulin Sensitivity Index (adjusted for BMI) (ISI)** | | | | | | | | |
| rs117643180 | A | C | 0.0231 | -0.1416 | 0.0221 | 1.58E-10 | 8.43E-04 | 45.24718218 |
| rs76619518 | C | G | 0.0722 | 0.0716 | 0.0122 | 4.91E-09 | 1.53E-03 | 81.96415789 |
| rs186333694 | A | G | 0.0742 | 0.0688 | 0.0121 | 1.44E-08 | 1.54E-03 | 82.64154762 |
| rs79953491 | A | G | 0.8808 | -0.0737 | 0.0098 | 5.19E-14 | 1.90E-03 | 102.0370129 |
| rs16959129 | A | G | 0.1401 | 0.0626 | 0.0092 | 9.12E-12 | 2.02E-03 | 108.6916007 |
| rs11128603 | A | G | 0.8595 | -0.0635 | 0.0091 | 2.64E-12 | 2.04E-03 | 109.8860139 |
| rs12753225 | C | G | 0.246 | 0.0466 | 0.0075 | 6.65E-10 | 2.48E-03 | 133.3283635 |
| rs10252039 | A | C | 0.2894 | -0.0384 | 0.007 | 4.86E-08 | 2.66E-03 | 142.851818 |
| rs1906937 | A | C | 0.2894 | -0.0384 | 0.007 | 4.86E-08 | 2.66E-03 | 142.851818 |
| rs459193 | A | G | 0.2815 | 0.0409 | 0.007 | 6.18E-09 | 2.66E-03 | 142.851818 |
| rs2295080 | T | G | 0.6961 | -0.0394 | 0.0069 | 1.34E-08 | 2.69E-03 | 144.9221342 |
| rs12454712 | T | C | 0.5858 | -0.0374 | 0.0068 | 4.42E-08 | 2.73E-03 | 147.0533421 |
| rs2972144 | A | G | 0.366 | 0.0644 | 0.0067 | 1.10E-21 | 2.77E-03 | 149.2481681 |
| rs6921742 | C | G | 0.6474 | -0.0419 | 0.0066 | 2.61E-10 | 2.82E-03 | 151.509504 |
| **Fasting insulin (FI)** | | | | | | | | |
| rs2780215 | A | G | 0.958 | 0.0392 | 0.0063 | 1.06E-09 | 1.05E-03 | 158.7280565 |
| rs116141873 | T | G | 0.032 | 0.0428 | 0.0059 | 1.42E-11 | 1.12E-03 | 169.4892807 |
| rs73690174 | T | C | 0.966 | -0.0377 | 0.0058 | 1.64E-09 | 1.14E-03 | 172.4115097 |
| rs12692462 | A | G | 0.96 | -0.0334 | 0.0058 | 7.60E-09 | 1.14E-03 | 172.4115097 |
| rs118164457 | T | C | 0.963 | -0.0345 | 0.0057 | 3.86E-10 | 1.16E-03 | 175.436273 |
| rs7699695 | T | C | 0.963 | -0.0323 | 0.0056 | 4.35E-09 | 1.18E-03 | 178.5690636 |
| rs62271373 | A | T | 0.059 | 0.0256 | 0.0048 | 1.60E-08 | 1.38E-03 | 208.3305742 |
| rs76619518 | C | G | 0.057 | -0.0273 | 0.0039 | 6.01E-11 | 1.70E-03 | 256.4068605 |
| rs2845885 | T | C | 0.931 | -0.0204 | 0.0039 | 1.18E-08 | 1.70E-03 | 256.4068605 |
| rs11727676 | T | C | 0.916 | -0.0203 | 0.0039 | 2.90E-08 | 1.70E-03 | 256.4068605 |
| rs186333694 | A | G | 0.049 | -0.0262 | 0.0038 | 7.16E-11 | 1.74E-03 | 263.1544095 |
| rs75179845 | T | C | 0.921 | -0.0216 | 0.0035 | 6.05E-11 | 1.89E-03 | 285.7105017 |
| rs6972708 | A | G | 0.083 | 0.0254 | 0.0032 | 4.00E-19 | 2.07E-03 | 312.4958613 |
| rs6039413 | A | G | 0.075 | 0.0264 | 0.0032 | 3.83E-20 | 2.07E-03 | 312.4958613 |
| rs10906092 | C | G | 0.129 | 0.0212 | 0.0031 | 1.87E-10 | 2.13E-03 | 322.5763729 |
| rs17331151 | T | C | 0.106 | -0.0162 | 0.0031 | 1.52E-08 | 2.13E-03 | 322.5763729 |
| rs9333447 | D | I | 0.133 | -0.0186 | 0.0031 | 3.53E-10 | 2.13E-03 | 322.5763729 |
| rs6014954 | T | C | 0.847 | 0.0181 | 0.003 | 1.09E-10 | 2.20E-03 | 333.3289187 |
| rs112328127 | A | C | 0.137 | 0.0219 | 0.003 | 2.93E-11 | 2.20E-03 | 333.3289187 |
| rs17036126 | T | C | 0.129 | 0.0209 | 0.003 | 1.28E-10 | 2.20E-03 | 333.3289187 |
| rs6005413 | A | G | 0.788 | 0.017 | 0.0029 | 6.16E-11 | 2.28E-03 | 344.8230193 |
| rs79046806 | A | G | 0.862 | -0.0224 | 0.0029 | 3.91E-13 | 2.28E-03 | 344.8230193 |
| rs35000407 | T | G | 0.883 | 0.0258 | 0.0028 | 1.50E-21 | 2.36E-03 | 357.1381272 |
| rs16959129 | A | G | 0.119 | -0.0248 | 0.0028 | 9.97E-21 | 2.36E-03 | 357.1381272 |
| rs13235110 | T | C | 0.208 | -0.016 | 0.0027 | 8.37E-10 | 2.45E-03 | 370.3654652 |
| rs5017305 | A | T | 0.236 | 0.0137 | 0.0026 | 1.07E-08 | 2.54E-03 | 384.6102908 |
| rs1045274 | A | T | 0.236 | 0.0137 | 0.0026 | 1.07E-08 | 2.54E-03 | 384.6102908 |
| rs1645364 | D | I | 0.773 | 0.0156 | 0.0026 | 3.06E-11 | 2.54E-03 | 384.6102908 |
| rs17002085 | T | C | 0.166 | 0.0179 | 0.0026 | 1.08E-14 | 2.54E-03 | 384.6102908 |
| rs74969426 | A | G | 0.796 | 0.0147 | 0.0026 | 8.32E-09 | 2.54E-03 | 384.6102908 |
| rs28613114 | A | G | 0.781 | 0.0188 | 0.0026 | 9.06E-13 | 2.54E-03 | 384.6102908 |
| rs34456785 | A | G | 0.831 | 0.0164 | 0.0026 | 5.26E-10 | 2.54E-03 | 384.6102908 |
| rs115838148 | C | G | 0.197 | -0.0147 | 0.0025 | 4.09E-10 | 2.64E-03 | 399.9947024 |
| rs13258890 | T | C | 0.748 | 0.0128 | 0.0025 | 2.77E-08 | 2.64E-03 | 399.9947024 |
| rs12454712 | T | C | 0.602 | 0.0142 | 0.0025 | 1.78E-09 | 2.64E-03 | 399.9947024 |
| rs860598 | A | G | 0.824 | 0.0177 | 0.0025 | 6.88E-12 | 2.64E-03 | 399.9947024 |
| rs117502478 | C | G | 0.79 | 0.0148 | 0.0025 | 1.20E-08 | 2.64E-03 | 399.9947024 |
| rs76850611 | T | G | 0.199 | 0.0114 | 0.0023 | 2.10E-08 | 2.87E-03 | 434.7768505 |
| rs2303623 | A | C | 0.774 | 0.015 | 0.0023 | 8.05E-12 | 2.87E-03 | 434.7768505 |
| rs10968208 | A | G | 0.226 | -0.0149 | 0.0023 | 4.95E-11 | 2.87E-03 | 434.7768505 |
| rs34748465 | T | C | 0.27 | 0.0133 | 0.0023 | 1.75E-09 | 2.87E-03 | 434.7768505 |
| rs11708067 | A | G | 0.823 | -0.0135 | 0.0023 | 1.30E-09 | 2.87E-03 | 434.7768505 |
| rs112298294 | T | C | 0.282 | -0.0135 | 0.0023 | 1.17E-09 | 2.87E-03 | 434.7768505 |
| rs12000334 | D | I | 0.259 | -0.012 | 0.0023 | 1.50E-08 | 2.87E-03 | 434.7768505 |
| rs12354616 | A | G | 0.784 | -0.011 | 0.0022 | 3.55E-08 | 3.00E-03 | 454.5394346 |
| rs7237968 | A | C | 0.306 | -0.0115 | 0.0022 | 1.23E-08 | 3.00E-03 | 454.5394346 |
| rs4572338 | D | I | 0.499 | -0.02 | 0.0022 | 1.33E-22 | 3.00E-03 | 454.5394346 |
| rs10865959 | C | G | 0.3 | 0.0138 | 0.0022 | 1.99E-08 | 3.00E-03 | 454.5394346 |
| rs79830100 | A | G | 0.61 | -0.0125 | 0.0021 | 2.12E-09 | 3.14E-03 | 476.1841696 |
| rs985722 | T | C | 0.363 | -0.012 | 0.0021 | 1.88E-08 | 3.14E-03 | 476.1841696 |
| rs7235514 | T | G | 0.511 | 0.0113 | 0.0021 | 4.13E-08 | 3.14E-03 | 476.1841696 |
| rs2830229 | A | C | 0.727 | 0.0159 | 0.0021 | 3.78E-15 | 3.14E-03 | 476.1841696 |
| rs114974628 | T | C | 0.657 | -0.0127 | 0.0021 | 6.65E-11 | 3.14E-03 | 476.1841696 |
| rs564640 | T | C | 0.34 | -0.0121 | 0.0021 | 4.89E-09 | 3.14E-03 | 476.1841696 |
| rs459193 | A | G | 0.285 | -0.0181 | 0.0021 | 1.12E-18 | 3.14E-03 | 476.1841696 |
| rs6545507 | A | G | 0.273 | -0.0175 | 0.0021 | 1.03E-17 | 3.14E-03 | 476.1841696 |
| rs34862974 | T | C | 0.362 | -0.0114 | 0.0021 | 1.26E-08 | 3.14E-03 | 476.1841696 |
| rs12110345 | C | G | 0.726 | 0.0127 | 0.0021 | 3.45E-08 | 3.14E-03 | 476.1841696 |
| rs2147977 | T | C | 0.34 | -0.012 | 0.0021 | 6.43E-09 | 3.14E-03 | 476.1841696 |
| rs7903146 | T | C | 0.307 | -0.0116 | 0.0021 | 1.24E-09 | 3.14E-03 | 476.1841696 |
| rs1009780 | T | G | 0.364 | -0.0115 | 0.0021 | 6.51E-09 | 3.14E-03 | 476.1841696 |
| rs11759749 | A | G | 0.653 | -0.0128 | 0.002 | 1.14E-09 | 3.30E-03 | 499.9933781 |
| rs4865796 | A | G | 0.707 | 0.0165 | 0.002 | 7.33E-17 | 3.30E-03 | 499.9933781 |
| rs4662685 | T | C | 0.273 | 0.0113 | 0.002 | 2.39E-09 | 3.30E-03 | 499.9933781 |
| rs1147272 | D | I | 0.512 | -0.0123 | 0.002 | 4.80E-11 | 3.30E-03 | 499.9933781 |
| rs6855363 | T | C | 0.653 | 0.0125 | 0.002 | 4.04E-08 | 3.30E-03 | 499.9933781 |
| rs6674544 | A | G | 0.574 | 0.0177 | 0.002 | 6.97E-21 | 3.30E-03 | 499.9933781 |
| rs77866501 | A | G | 0.566 | 0.0215 | 0.002 | 3.99E-27 | 3.30E-03 | 499.9933781 |
| rs13250832 | T | C | 0.716 | 0.0142 | 0.002 | 8.45E-13 | 3.30E-03 | 499.9933781 |
| rs2108349 | A | G | 0.686 | -0.0115 | 0.002 | 1.13E-08 | 3.30E-03 | 499.9933781 |
| rs7073353 | T | C | 0.623 | -0.0107 | 0.0019 | 1.72E-09 | 3.47E-03 | 526.308819 |
| rs567098617 | A | G | 0.555 | 0.02 | 0.0019 | 9.93E-29 | 3.47E-03 | 526.308819 |
| rs731839 | A | G | 0.658 | -0.0121 | 0.0019 | 3.87E-11 | 3.47E-03 | 526.308819 |
| rs6905288 | A | G | 0.602 | 0.0112 | 0.0019 | 7.75E-09 | 3.47E-03 | 526.308819 |
| rs1206760 | A | G | 0.522 | -0.0112 | 0.0019 | 8.82E-10 | 3.47E-03 | 526.308819 |
| rs11872375 | A | G | 0.57 | -0.0112 | 0.0019 | 4.84E-08 | 3.47E-03 | 526.308819 |
| rs1355406 | T | C | 0.538 | 0.0125 | 0.0019 | 1.24E-11 | 3.47E-03 | 526.308819 |
| rs10099692 | T | C | 0.549 | -0.0105 | 0.0019 | 2.32E-08 | 3.47E-03 | 526.308819 |
| rs10050393 | T | C | 0.54 | 0.009 | 0.0019 | 4.84E-08 | 3.47E-03 | 526.308819 |
| rs13389219 | T | C | 0.409 | -0.0199 | 0.0019 | 5.84E-28 | 3.47E-03 | 526.308819 |
| rs2943646 | A | G | 0.377 | -0.025 | 0.0019 | 8.47E-39 | 3.47E-03 | 526.308819 |
| rs7012814 | A | G | 0.471 | -0.0219 | 0.0019 | 8.34E-30 | 3.47E-03 | 526.308819 |
| rs71534286 | A | G | 0.413 | 0.0104 | 0.0019 | 1.68E-09 | 3.47E-03 | 526.308819 |
| rs2118193 | A | G | 0.5 | -0.0118 | 0.0019 | 2.71E-10 | 3.47E-03 | 526.308819 |
| rs9884482 | T | C | 0.608 | -0.0125 | 0.0019 | 2.88E-11 | 3.47E-03 | 526.308819 |
| rs972283 | A | G | 0.456 | -0.0105 | 0.0019 | 1.09E-08 | 3.47E-03 | 526.308819 |
| rs6921742 | C | G | 0.623 | 0.02 | 0.0019 | 6.98E-27 | 3.47E-03 | 526.308819 |
| rs1351394 | T | C | 0.471 | -0.0111 | 0.0018 | 2.71E-09 | 3.67E-03 | 555.5481978 |
| rs3775380 | A | G | 0.5 | -0.0119 | 0.0018 | 1.48E-11 | 3.67E-03 | 555.5481978 |
| rs1474696 | A | G | 0.524 | -0.0147 | 0.0018 | 3.02E-16 | 3.67E-03 | 555.5481978 |
| **Proinsulin (PROI)** | | | | | | | | |
| rs10186386 | A | T | 0.9757 | -0.2177 | 0.0351 | 5.72E-10 | 6.21E-04 | 28.48878604 |
| rs148072650 | A | G | 0.9757 | -0.2177 | 0.0351 | 5.72E-10 | 6.21E-04 | 28.48878604 |
| rs550333 | T | C | 0.9856 | -0.2343 | 0.0351 | 2.55E-11 | 6.21E-04 | 28.48878604 |
| rs9330360 | A | G | 0.9681 | -0.2186 | 0.0345 | 2.40E-10 | 6.32E-04 | 28.98424319 |
| rs57866780 | T | G | 0.974 | -0.1889 | 0.0332 | 1.27E-08 | 6.56E-04 | 30.11916837 |
| rs2535388 | A | G | 0.0293 | 0.2098 | 0.033 | 2.03E-10 | 6.60E-04 | 30.30170879 |
| rs7833402 | A | G | 0.015 | 0.3728 | 0.0322 | 4.94E-31 | 6.77E-04 | 31.05454627 |
| rs11697874 | A | G | 0.0297 | 0.2022 | 0.0321 | 2.92E-10 | 6.79E-04 | 31.15128941 |
| rs61741902 | A | G | 0.0131 | 0.4688 | 0.0319 | 5.83E-49 | 6.83E-04 | 31.3465953 |
| rs17134857 | T | C | 0.0294 | 0.1995 | 0.0318 | 3.42E-10 | 6.85E-04 | 31.4451695 |
| rs117322158 | T | C | 0.0222 | 0.2705 | 0.0264 | 1.20E-24 | 8.25E-04 | 37.87713598 |
| rs6442708 | C | G | 0.0222 | 0.2412 | 0.0248 | 2.09E-22 | 8.78E-04 | 40.32082218 |
| rs142927921 | A | G | 0.025 | 0.1632 | 0.0247 | 3.70E-11 | 8.82E-04 | 40.48406437 |
| rs117883954 | A | G | 0.0271 | 0.137 | 0.024 | 1.08E-08 | 9.08E-04 | 41.66484958 |
| rs80088612 | C | G | 0.0264 | 0.1541 | 0.0215 | 8.05E-13 | 1.01E-03 | 46.50959953 |
| rs12885207 | T | C | 0.0505 | -0.1698 | 0.0188 | 1.55E-19 | 1.16E-03 | 53.18916968 |
| rs10231448 | T | C | 0.0641 | -0.2379 | 0.0183 | 9.52E-39 | 1.19E-03 | 54.64242568 |
| rs74920406 | T | C | 0.0402 | -0.1464 | 0.018 | 3.71E-16 | 1.21E-03 | 55.55313278 |
| rs6833249 | T | C | 0.0396 | -0.1056 | 0.0179 | 3.41E-09 | 1.22E-03 | 55.86348547 |
| rs75457267 | T | C | 0.0444 | -0.1014 | 0.017 | 2.22E-09 | 1.28E-03 | 58.82096412 |
| rs8020595 | T | C | 0.062 | -0.2828 | 0.0161 | 1.00E-68 | 1.35E-03 | 62.10909254 |
| rs62134815 | T | C | 0.9379 | 0.1423 | 0.0151 | 5.08E-21 | 1.44E-03 | 66.22227748 |
| rs2172011 | T | G | 0.9387 | 0.0965 | 0.0143 | 1.57E-11 | 1.52E-03 | 69.92702028 |
| rs6499736 | T | C | 0.0723 | -0.1328 | 0.0141 | 4.91E-21 | 1.54E-03 | 70.91889291 |
| rs149766034 | A | G | 0.0642 | 0.0876 | 0.0139 | 3.00E-10 | 1.57E-03 | 71.93930863 |
| rs9883138 | A | G | 0.0806 | 0.1013 | 0.0135 | 6.25E-14 | 1.61E-03 | 74.0708437 |
| rs77839149 | A | T | 0.8986 | 0.1153 | 0.0121 | 1.37E-21 | 1.80E-03 | 82.64102396 |
| rs4819130 | T | G | 0.084 | 0.0838 | 0.0121 | 4.01E-12 | 1.80E-03 | 82.64102396 |
| rs61853599 | T | C | 0.8782 | 0.0613 | 0.0109 | 1.67E-08 | 2.00E-03 | 91.73911835 |
| rs728505 | A | G | 0.121 | -0.0613 | 0.0109 | 1.67E-08 | 2.00E-03 | 91.73911835 |
| rs8029218 | T | C | 0.873 | 0.0643 | 0.0106 | 1.13E-09 | 2.05E-03 | 94.33550849 |
| rs56252324 | A | C | 0.8735 | 0.061 | 0.0105 | 5.42E-09 | 2.07E-03 | 95.2339419 |
| rs34890839 | A | G | 0.8746 | 0.0604 | 0.0105 | 7.63E-09 | 2.07E-03 | 95.2339419 |
| rs143050036 | A | G | 0.8634 | -0.0583 | 0.0103 | 1.30E-08 | 2.11E-03 | 97.08314466 |
| rs58297911 | C | G | 0.1384 | 0.056 | 0.0103 | 4.72E-08 | 2.11E-03 | 97.08314466 |
| rs10452028 | A | T | 0.1501 | 0.0839 | 0.0099 | 3.36E-17 | 2.20E-03 | 101.005696 |
| rs7531586 | A | G | 0.1326 | 0.0729 | 0.0097 | 7.42E-14 | 2.24E-03 | 103.0882876 |
| rs4508606 | A | G | 0.1394 | 0.0556 | 0.0097 | 1.16E-08 | 2.24E-03 | 103.0882876 |
| rs1060043 | T | C | 0.856 | -0.1009 | 0.0095 | 3.95E-26 | 2.29E-03 | 105.2585674 |
| rs16876519 | A | G | 0.8509 | 0.0526 | 0.0095 | 3.55E-08 | 2.29E-03 | 105.2585674 |
| rs12712928 | C | G | 0.1612 | 0.091 | 0.0095 | 1.48E-21 | 2.29E-03 | 105.2585674 |
| rs11211230 | A | G | 0.1508 | 0.0866 | 0.0094 | 4.61E-20 | 2.31E-03 | 106.3783394 |
| rs114923365 | A | G | 0.8479 | -0.0866 | 0.0094 | 4.61E-20 | 2.31E-03 | 106.3783394 |
| rs118147297 | A | C | 0.8454 | -0.0859 | 0.0093 | 3.67E-20 | 2.34E-03 | 107.5221925 |
| rs2977105 | T | C | 0.1762 | -0.0558 | 0.0091 | 1.01E-09 | 2.39E-03 | 109.8853176 |
| rs13092582 | A | G | 0.1825 | 0.0659 | 0.0089 | 1.62E-13 | 2.44E-03 | 112.3546506 |
| rs34859455 | A | G | 0.1825 | 0.0659 | 0.0089 | 1.62E-13 | 2.44E-03 | 112.3546506 |
| rs79206774 | A | C | 0.1776 | 0.0839 | 0.0089 | 5.91E-21 | 2.44E-03 | 112.3546506 |
| rs1460464 | A | G | 0.2004 | -0.0878 | 0.0086 | 2.56E-24 | 2.53E-03 | 116.2739988 |
| rs77464186 | A | C | 0.8079 | -0.2587 | 0.0085 | 1.00E-200 | 2.56E-03 | 117.6419282 |
| rs144013274 | A | G | 0.7997 | 0.0585 | 0.0083 | 2.10E-12 | 2.62E-03 | 120.4766735 |
| rs11139605 | T | C | 0.2432 | -0.0595 | 0.0082 | 4.62E-13 | 2.65E-03 | 121.9459012 |
| rs8063370 | C | G | 0.7176 | -0.0736 | 0.0082 | 3.54E-19 | 2.65E-03 | 121.9459012 |
| rs11766164 | A | G | 0.2376 | 0.048 | 0.0081 | 3.41E-09 | 2.68E-03 | 123.4514062 |
| rs36118066 | A | G | 0.7745 | 0.0899 | 0.0081 | 1.76E-28 | 2.68E-03 | 123.4514062 |
| rs111925767 | T | G | 0.2328 | 0.049 | 0.0081 | 1.61E-09 | 2.68E-03 | 123.4514062 |
| rs10507349 | A | G | 0.2215 | -0.0482 | 0.008 | 1.85E-09 | 2.72E-03 | 124.9945487 |
| rs150182635 | T | C | 0.2376 | 0.048 | 0.008 | 2.16E-09 | 2.72E-03 | 124.9945487 |
| rs7810127 | A | G | 0.7551 | 0.0448 | 0.0079 | 1.53E-08 | 2.75E-03 | 126.5767582 |
| rs4790075 | T | C | 0.7414 | 0.0641 | 0.0078 | 2.40E-16 | 2.79E-03 | 128.1995372 |
| rs614864 | A | G | 0.2558 | -0.0483 | 0.0078 | 6.45E-10 | 2.79E-03 | 128.1995372 |
| rs77408854 | T | C | 0.7643 | 0.0515 | 0.0078 | 4.45E-11 | 2.79E-03 | 128.1995372 |
| rs10501320 | C | G | 0.243 | -0.2144 | 0.0078 | 1.27E-165 | 2.79E-03 | 128.1995372 |
| rs155438 | C | G | 0.6018 | -0.0519 | 0.0078 | 3.15E-11 | 2.79E-03 | 128.1995372 |
| rs58909947 | T | C | 0.7424 | 0.1548 | 0.0077 | 1.52E-89 | 2.82E-03 | 129.8644662 |
| rs7903146 | T | C | 0.2604 | 0.1014 | 0.0077 | 1.87E-39 | 2.82E-03 | 129.8644662 |
| rs10059071 | T | C | 0.7342 | 0.047 | 0.0076 | 6.70E-10 | 2.86E-03 | 131.5732092 |
| rs3849691 | A | G | 0.285 | -0.0512 | 0.0076 | 1.76E-11 | 2.86E-03 | 131.5732092 |
| rs115652575 | A | G | 0.2717 | -0.1258 | 0.0076 | 2.52E-61 | 2.86E-03 | 131.5732092 |
| rs77467424 | T | G | 0.7243 | 0.1313 | 0.0075 | 2.10E-68 | 2.90E-03 | 133.3275187 |
| rs6235 | C | G | 0.7244 | -0.1219 | 0.0075 | 3.26E-59 | 2.90E-03 | 133.3275187 |
| rs2302783 | T | C | 0.2817 | -0.0424 | 0.0074 | 1.06E-08 | 2.94E-03 | 135.1292419 |
| rs11941759 | A | G | 0.669 | -0.0483 | 0.0074 | 7.15E-11 | 2.94E-03 | 135.1292419 |
| rs529923 | T | C | 0.3742 | 0.0998 | 0.0074 | 2.45E-41 | 2.94E-03 | 135.1292419 |
| rs368476 | A | G | 0.6527 | 0.0694 | 0.0074 | 7.62E-21 | 2.94E-03 | 135.1292419 |
| rs12346799 | A | T | 0.6602 | 0.0429 | 0.0074 | 7.09E-09 | 2.94E-03 | 135.1292419 |
| rs6066846 | A | G | 0.3098 | 0.0698 | 0.0073 | 1.30E-21 | 2.98E-03 | 136.9803274 |
| rs3183175 | T | C | 0.3053 | -0.04 | 0.0073 | 4.44E-08 | 2.98E-03 | 136.9803274 |
| rs6976645 | A | G | 0.3052 | -0.0405 | 0.0073 | 3.01E-08 | 2.98E-03 | 136.9803274 |
| rs77542522 | T | C | 0.7103 | -0.0856 | 0.0073 | 1.12E-31 | 2.98E-03 | 136.9803274 |
| rs7116599 | C | G | 0.3353 | -0.0643 | 0.0073 | 1.40E-18 | 2.98E-03 | 136.9803274 |
| rs181685789 | A | G | 0.68 | -0.1079 | 0.0073 | 2.57E-49 | 2.98E-03 | 136.9803274 |
| rs2404224 | A | G | 0.6879 | 0.0454 | 0.0072 | 3.00E-10 | 3.02E-03 | 138.8828319 |
| rs13103660 | T | C | 0.6666 | -0.0516 | 0.0072 | 8.13E-13 | 3.02E-03 | 138.8828319 |
| rs71491746 | T | C | 0.3541 | -0.0439 | 0.0072 | 1.12E-09 | 3.02E-03 | 138.8828319 |
| rs826415 | T | G | 0.6744 | 0.0437 | 0.0072 | 1.34E-09 | 3.02E-03 | 138.8828319 |
| rs4300038 | A | G | 0.3361 | -0.0943 | 0.0072 | 4.11E-39 | 3.02E-03 | 138.8828319 |
| rs79829102 | A | G | 0.3752 | 0.0775 | 0.0072 | 5.78E-27 | 3.02E-03 | 138.8828319 |
| rs6702126 | A | G | 0.3543 | -0.0442 | 0.0072 | 8.66E-10 | 3.02E-03 | 138.8828319 |
| rs78641829 | A | G | 0.5441 | 0.045 | 0.0071 | 2.41E-10 | 3.06E-03 | 140.8389282 |
| rs7139043 | T | G | 0.3244 | 0.071 | 0.0071 | 1.66E-23 | 3.06E-03 | 140.8389282 |
| rs1217636 | A | C | 0.336 | -0.1333 | 0.0071 | 1.66E-78 | 3.06E-03 | 140.8389282 |
| rs10004088 | T | C | 0.4143 | 0.0567 | 0.0071 | 1.48E-15 | 3.06E-03 | 140.8389282 |
| rs2470392 | A | G | 0.6459 | 0.0438 | 0.007 | 4.03E-10 | 3.11E-03 | 142.8509129 |
| rs114776512 | A | G | 0.339 | 0.076 | 0.007 | 2.00E-27 | 3.11E-03 | 142.8509129 |
| rs4131631 | T | C | 0.6309 | -0.1019 | 0.007 | 6.07E-48 | 3.11E-03 | 142.8509129 |
| rs147126736 | A | G | 0.6463 | 0.0479 | 0.007 | 8.02E-12 | 3.11E-03 | 142.8509129 |
| rs553768 | A | G | 0.3538 | -0.0431 | 0.007 | 7.60E-10 | 3.11E-03 | 142.8509129 |
| rs77769171 | C | G | 0.6461 | 0.0429 | 0.007 | 9.10E-10 | 3.11E-03 | 142.8509129 |
| rs55811236 | A | C | 0.3538 | -0.0471 | 0.007 | 1.77E-11 | 3.11E-03 | 142.8509129 |
| rs4803986 | A | G | 0.6589 | -0.0648 | 0.007 | 2.23E-20 | 3.11E-03 | 142.8509129 |
| rs4941564 | A | G | 0.3577 | 0.0673 | 0.007 | 7.41E-22 | 3.11E-03 | 142.8509129 |
| rs7160311 | T | C | 0.3399 | 0.0761 | 0.007 | 1.71E-27 | 3.11E-03 | 142.8509129 |
| rs76026094 | A | G | 0.6609 | -0.076 | 0.007 | 2.00E-27 | 3.11E-03 | 142.8509129 |
| rs7478446 | T | C | 0.4298 | -0.0603 | 0.007 | 7.40E-18 | 3.11E-03 | 142.8509129 |
| rs80019657 | C | G | 0.3708 | 0.1023 | 0.007 | 2.63E-48 | 3.11E-03 | 142.8509129 |
| rs57749251 | T | C | 0.5779 | -0.062 | 0.0069 | 2.67E-19 | 3.15E-03 | 144.9212159 |
| rs7117789 | T | C | 0.3982 | 0.0454 | 0.0069 | 4.81E-11 | 3.15E-03 | 144.9212159 |
| rs1859437 | T | C | 0.6385 | 0.1265 | 0.0069 | 5.23E-75 | 3.15E-03 | 144.9212159 |
| rs62049757 | A | G | 0.5923 | -0.0666 | 0.0069 | 5.02E-22 | 3.15E-03 | 144.9212159 |
| rs310612 | T | C | 0.4054 | 0.0667 | 0.0069 | 4.36E-22 | 3.15E-03 | 144.9212159 |
| rs1911924 | A | G | 0.5698 | 0.0549 | 0.0069 | 1.82E-15 | 3.15E-03 | 144.9212159 |
| rs2796441 | A | G | 0.4121 | -0.0514 | 0.0069 | 9.63E-14 | 3.15E-03 | 144.9212159 |
| rs72917944 | C | G | 0.4302 | -0.0547 | 0.0069 | 2.30E-15 | 3.15E-03 | 144.9212159 |
| rs11691390 | A | G | 0.4771 | 0.0698 | 0.0068 | 1.04E-24 | 3.20E-03 | 147.0524103 |
| rs1019627 | T | C | 0.5712 | 0.0592 | 0.0068 | 3.21E-18 | 3.20E-03 | 147.0524103 |
| rs78853795 | T | C | 0.51 | -0.0575 | 0.0068 | 2.82E-17 | 3.20E-03 | 147.0524103 |
| rs549715518 | A | C | 0.5313 | 0.0462 | 0.0068 | 1.10E-11 | 3.20E-03 | 147.0524103 |
| rs76767088 | A | G | 0.5738 | 0.0624 | 0.0068 | 4.55E-20 | 3.20E-03 | 147.0524103 |
| rs117767193 | A | C | 0.4916 | 0.0621 | 0.0068 | 6.84E-20 | 3.20E-03 | 147.0524103 |
| rs114023299 | T | C | 0.5112 | -0.065 | 0.0068 | 1.22E-21 | 3.20E-03 | 147.0524103 |
| rs10784277 | T | C | 0.5112 | -0.065 | 0.0068 | 1.22E-21 | 3.20E-03 | 147.0524103 |
| rs11871618 | A | G | 0.5259 | 0.0404 | 0.0068 | 2.86E-09 | 3.20E-03 | 147.0524103 |
| rs12714515 | A | T | 0.5488 | -0.0497 | 0.0068 | 2.73E-13 | 3.20E-03 | 147.0524103 |
| rs1184466 | T | C | 0.5494 | -0.0492 | 0.0068 | 4.71E-13 | 3.20E-03 | 147.0524103 |
| rs11989394 | A | G | 0.3945 | 0.0666 | 0.0068 | 1.22E-22 | 3.20E-03 | 147.0524103 |
| rs12708529 | A | T | 0.5476 | -0.0489 | 0.0068 | 6.51E-13 | 3.20E-03 | 147.0524103 |
| rs9595639 | C | G | 0.5713 | 0.0617 | 0.0067 | 3.30E-20 | 3.24E-03 | 149.2472224 |
| rs2762488 | A | G | 0.4403 | 0.0569 | 0.0067 | 2.03E-17 | 3.24E-03 | 149.2472224 |
| rs1595979 | A | G | 0.5364 | 0.0877 | 0.0067 | 3.79E-39 | 3.24E-03 | 149.2472224 |
| rs187278176 | T | C | 0.5217 | 0.0875 | 0.0067 | 5.61E-39 | 3.24E-03 | 149.2472224 |
| rs117600549 | T | C | 0.4494 | 0.0567 | 0.0067 | 2.62E-17 | 3.24E-03 | 149.2472224 |
| rs7097350 | T | C | 0.5158 | 0.0475 | 0.0067 | 1.35E-12 | 3.24E-03 | 149.2472224 |
| rs60804270 | C | G | 0.4631 | -0.0875 | 0.0067 | 5.61E-39 | 3.24E-03 | 149.2472224 |
| rs11856307 | A | C | 0.5418 | 0.0886 | 0.0067 | 6.41E-40 | 3.24E-03 | 149.2472224 |
| rs34836887 | C | G | 0.5531 | 0.1068 | 0.0067 | 3.34E-57 | 3.24E-03 | 149.2472224 |
| rs114610484 | A | C | 0.4738 | -0.0431 | 0.0067 | 1.25E-10 | 3.24E-03 | 149.2472224 |
| rs138515317 | T | C | 0.5462 | -0.0569 | 0.0067 | 2.03E-17 | 3.24E-03 | 149.2472224 |
| **Parkinson's disease (PD)** | | | | | | | | |
| rs144814361 | T | C | 0.0174 | 0.4411 | 0.068 | 9.07E-11 | 3.05E-05 | 14.70582142 |
| rs75505347 | T | C | 0.0195 | 0.3917 | 0.0674 | 6.12E-09 | 3.07E-05 | 14.83673378 |
| rs35749011 | A | G | 0.0191 | 0.7508 | 0.0659 | 5.02E-30 | 3.14E-05 | 15.17444396 |
| rs35265698 | G | C | 0.1547 | -0.2 | 0.0303 | 3.93E-11 | 6.87E-05 | 33.00316299 |
| rs75646569 | G | T | 0.1117 | 0.1916 | 0.0266 | 5.62E-13 | 7.79E-05 | 37.59382921 |
| rs58879558 | C | T | 0.2229 | -0.2383 | 0.025 | 1.36E-21 | 8.29E-05 | 39.99983428 |
| rs4613239 | G | C | 0.1326 | 0.1784 | 0.0248 | 6.21E-13 | 8.35E-05 | 40.32241358 |
| rs823106 | C | G | 0.8488 | -0.1492 | 0.0239 | 4.10E-10 | 8.67E-05 | 41.84083083 |
| rs34311866 | C | T | 0.1958 | 0.2272 | 0.0231 | 7.97E-23 | 8.97E-05 | 43.28986393 |
| rs10513789 | G | T | 0.1826 | -0.1596 | 0.0219 | 3.18E-13 | 9.46E-05 | 45.66191127 |
| rs7695720 | C | A | 0.2091 | -0.1255 | 0.0208 | 1.53E-09 | 9.96E-05 | 48.07672389 |
| rs4488803 | A | G | 0.3746 | -0.1136 | 0.0199 | 1.08E-08 | 1.04E-04 | 50.25104809 |
| rs4774417 | A | G | 0.7397 | 0.1052 | 0.0192 | 4.63E-08 | 1.08E-04 | 52.08311755 |
| rs620490 | G | T | 0.2762 | -0.1174 | 0.019 | 6.46E-10 | 1.09E-04 | 52.63136089 |
| rs12934900 | T | A | 0.6571 | 0.1215 | 0.0184 | 4.33E-11 | 1.13E-04 | 54.34760092 |
| rs10847864 | T | G | 0.3625 | 0.1274 | 0.0179 | 9.81E-13 | 1.16E-04 | 55.86569033 |
| rs329647 | C | G | 0.6662 | -0.1133 | 0.0178 | 1.94E-10 | 1.16E-04 | 56.17954252 |
| rs4588066 | A | G | 0.326 | 0.1046 | 0.0178 | 4.45E-09 | 1.16E-04 | 56.17954252 |
| rs356203 | T | C | 0.6169 | -0.2398 | 0.0178 | 3.01E-41 | 1.16E-04 | 56.17954252 |
| rs858295 | G | A | 0.3947 | -0.1039 | 0.0176 | 3.83E-09 | 1.18E-04 | 56.81794641 |
| rs6741007 | G | T | 0.4507 | -0.1233 | 0.0175 | 2.09E-12 | 1.18E-04 | 57.14262039 |
| rs10451230 | T | A | 0.565 | -0.096 | 0.0175 | 4.42E-08 | 1.18E-04 | 57.14262039 |
| rs4698412 | A | G | 0.553 | 0.1258 | 0.0168 | 7.05E-14 | 1.23E-04 | 59.52356291 |

SNP, single nucleotide polymorphism; EA, effect allele; OA, other allele; EAF, effect allele frequency; BETA, genetic effects of SNP on exposure; SE, standard error; P: P-value; PD, Parkinson's disease; UPDRS3, Unified Parkinson's Disease Rating Scale part III; MMSE, Mini-Mental State Examination; MoCA, Montreal Cognitive Assessment; R^2^ indicates the proportion of variance in exposure explained by each of the SNPs: 𝑅^2^ = [2 × BETA^2^ × (1 − EAF) × EAF] / [2 × BETA^2^ × (1 − EAF) × EAF + 2 × BETA^2^ ×SE × 𝑁 × (1 − EAF) × EAF]; F-statistic for each of SNPs was calculated using the formula: 𝑅^2^ × (𝑁 – k-1) / (1 − 𝑅^2^) × k; N, sample size; k, number of SNPs included in calculation.

**Table S2** Phenotypes significantly associated with instrumental variables of Diabetes Mellitus and Glycemic Traits identified by PhenoScanner.

| **SNP** | **EA** | **OA** | **Trait** | **P** | **PMID** |
| --- | --- | --- | --- | --- | --- |
| **Type 1 diabetes (T1D)** | | | | | |
| rs3184504 | C | T | Serum urate | 3.00E-11 | 24816252 |
| rs3184504 | C | T | Serum urate | 2.03E-11 | UKBB |
| rs3184504 | C | T | Past tobacco smoking | 1.00E-12 | 20228798 |
| rs3184504 | C | T | Smoking status: previous | 8.57E-13 | 27863252 |
| rs601338 | A | G | Alcohol intake frequency | 3.04E-17 | 27863252 |
| rs35327136 | A | C | Medication for pain relief, constipation, heartburn: paracetamol | 3.60E-26 | 24390342 |
| rs35327136 | A | C | Treatment with paracetamol | 3.00E-27 | 19430480 |
| **Type 2 diabetes (T2D)** | | | | | |
| rs1260326 | C | T | Serum urate | 5.90E-17 | 20884846 |
| rs1260326 | C | T | Serum urate | 7.66E-14 | 21768215 |
| rs1260326 | C | T | Serum urate | 1.25E-44 | 23263486 |
| rs1260326 | C | T | Uric acid | 1.82E-09 | 19503597 |
| rs1260326 | C | T | Serum urate | 1.31E-40 | 23263486 |
| rs1260326 | C | T | Gout | 2.00E-12 | 25646370 |
| rs1260326 | C | T | Gout | 7.00E-11 | 27899376 |
| rs1260326 | C | T | Urate levels | 1.00E-44 | 23263486 |
| rs6937438 | A | G | Serum urate | 4.09E-08 | 23263486 |
| rs6937438 | A | G | Serum urate | 4.09E-08 | 23263486 |
| rs2383205 | A | G | Medication for pain relief, constipation, heartburn: aspirin | 4.08E-12 | UKBB |
| rs2383205 | A | G | Treatment with aspirin | 2.81E-11 | UKBB |
| rs429358 | C | T | Frequency of stair climbing in last 4 weeks | 3.94E-08 | UKBB |
| rs429358 | C | T | Number of days or week of moderate physical activity 10+ minutes | 5.00E-08 | UKBB |
| **Glycated hemoglobin levels (HbA1c)** | | | | | |
| rs10774624 | A | G | Past tobacco smoking | 2.00E-09 | UKBB |
| **Fasting glucose (FG)** | | | | | |
| rs6598541 | A | G | Serum urate | 4.75E-15 | 23263486 |
| rs6598541 | A | G | Serum urate | 5.20E-13 | 23263486 |
| rs6598541 | A | G | Urate levels | 5.00E-15 | 23263486 |
| **Two-hour glucose (2hGlu)** | | | | | |
| rs1260326 | C | T | Serum urate | 5.90E-17 | 20884846 |
| rs1260326 | C | T | Serum urate | 7.66E-14 | 21768215 |
| rs1260326 | C | T | Serum urate | 1.25E-44 | 23263486 |
| rs1260326 | C | T | Uric acid | 1.82E-09 | 19503597 |
| rs1260326 | C | T | Serum urate | 1.31E-40 | 23263486 |
| rs1260326 | C | T | Gout | 2.00E-12 | 25646370 |
| rs1260326 | C | T | Gout | 7.00E-11 | 27899376 |
| rs1260326 | C | T | Renal overload gout | 5.00E-09 | 27899376 |
| rs1260326 | C | T | Urate levels | 1.00E-44 | 23263486 |
| rs1260326 | C | T | Self-reported gout | 3.83E-28 | UKBB |
| **Insulin fold change during an oral glucose tolerance test (adjusted for BMI) (IFC)** | | | | | |
| NA | NA | NA | NA | NA | NA |
| **Modified Stumvoll Insulin Sensitivity Index (adjusted for BMI)(ISI)** | | | | | |
| NA | NA | NA | NA | NA | NA |
| **Fasting insulin (FI)** | | | | | |
| rs1260326 | C | T | Serum urate | 5.90E-17 | 20884846 |
| rs1260326 | C | T | Serum urate | 7.66E-14 | 21768215 |
| rs1260326 | C | T | Serum urate | 1.25E-44 | 23263486 |
| rs1260326 | C | T | Uric acid | 1.82E-09 | 19503597 |
| rs1260326 | C | T | Serum urate | 1.31E-40 | 23263486 |
| rs1260326 | C | T | Gout | 2.00E-12 | 25646370 |
| rs1260326 | C | T | Gout | 7.00E-11 | 27899376 |
| rs1260326 | C | T | Renal overload gout | 5.00E-09 | 27899376 |
| rs1260326 | C | T | Urate levels | 1.00E-44 | 23263486 |
| rs1260326 | C | T | Self-reported gout | 3.83E-28 | UKBB |
| **Proinsulin (PROI)** | | | | | |
| NA | NA | NA | NA | NA | NA |
| **Parkinson's disease risk (PD risk)** | | | | | |
| NA | NA | NA | NA | NA | NA |

SNP, single nucleotide polymorphism; EA, effect allele; OA, other allele; P: P-value.

**Table S3** Results of the MR analysis and Sensitivity analysis in forward MR

| **Outcome** | **N  SNVs** | **MR  analysis** | | |  | **Heterogeneity  Test** | |  | **MR-Egger pleiotropy  Test** | |  | **MR PRESSO Test** |  | **Directionality  Test** | |
| --- | --- | --- | --- | --- | --- | --- | --- | --- | --- | --- | --- | --- | --- | --- | --- |
|  |  | **Method** | **Estimate (95% CI)** | ***P*** |  | **Q value** | ***P*** |  | **Egger intercept** | ***P*** |  | **Global Test *P*** |  | **Correct directionaliy** | ***P*** |
| **Type 1 diabetes (T1DM)** | | | | | | | | | | | | | | | |
| PD risk * | 68 | IVW | 0.9708(0.9466, 0.9956) | 0.0214 |  | 69.3901 | 0.3968 |  | 0.0002 | 0.9704 |  | 0.3885 |  | TRUE | 0.0000 |
|  |  | MR_Egger | 0.9702(0.9324, 1.0097) | 0.1420 |  | 69.3886 | 0.3640 |  |  |  |  |  |  |  |  |
|  |  | Weighted_median | 0.9454(0.9079, 0.9844) | 0.0065 |  |  |  |  |  |  |  |  |  |  |  |
| PD AAO | 49 | IVW | -0.0432(-0.2235, 0.1369) | 0.6379 |  | 36.5035 | 0.8875 |  | -0.0132 | 0.6908 |  | 0.8941 |  | TRUE | 0.0000 |
|  |  | MR_Egger | 0.0025(-0.2851, 0.2901) | 0.9864 |  | 36.3433 | 0.8697 |  |  |  |  |  |  |  |  |
|  |  | Weighted_median | 0.0098(-0.2592, 0.2788) | 0.9430 |  |  |  |  |  |  |  |  |  |  |  |
| UPDRS3 | 29 | IVW | 0.0081(-0.0347, 0.0508) | 0.7120 |  | 6.2855 | 1.0000 |  | 0.0059 | 0.5376 |  | 1.0000 |  | TRUE | 0.6823 |
|  |  | MR_Egger | -0.0084(-0.0755, 0.0587) | 0.8078 |  | 5.8955 | 1.0000 |  |  |  |  |  |  |  |  |
|  |  | Weighted_median | 0.0077(-0.0467, 0.0621) | 0.7815 |  |  |  |  |  |  |  |  |  |  |  |
| MMSE | 31 | IVW | -0.0502(-0.1265, 0.0261) | 0.1969 |  | 8.1621 | 1.0000 |  | 0.0018 | 0.9106 |  | 1.0000 |  | TRUE | 0.0654 |
|  |  | MR_Egger | -0.0556(-0.1765, 0.0653) | 0.3745 |  | 8.1492 | 1.0000 |  |  |  |  |  |  |  |  |
|  |  | Weighted_median | -0.0998(-0.2031, 0.0035) | 0.0582 |  |  |  |  |  |  |  |  |  |  |  |
| MOCA | 23 | IVW | -0.0983(-0.3123, 0.1157) | 0.3678 |  | 7.9993 | 0.9972 |  | 0.0037 | 0.9445 |  | 0.9951 |  | TRUE | 0.8692 |
|  |  | MR_Egger | -0.1081(-0.4550, 0.2387) | 0.5477 |  | 7.9943 | 0.9952 |  |  |  |  |  |  |  |  |
|  |  | Weighted_median | -0.1925(-0.4825, 0.0975) | 0.1932 |  |  |  |  |  |  |  |  |  |  |  |
| **Type 2 diabetes (T2DM)** | | | | | | | | | | | | | | | |
| PD risk | 155 | IVW | 1.0310(0.9733, 1.0921) | 0.2989 |  | 195.3639 | 0.0135 |  | -0.0072 | 0.1128 |  | 0.0135 |  | TRUE | 0.0000 |
|  |  | MR_Egger | 1.1330(0.9956, 1.2894) | 0.0603 |  | 192.1686 | 0.0174 |  |  |  |  |  |  |  |  |
|  |  | Weighted_median | 1.0352(0.9454, 1.1336) | 0.4544 |  |  |  |  |  |  |  |  |  |  |  |
| PD AAO | 120 | IVW | 0.0784(-0.2882, 0.4450) | 0.6750 |  | 67.9232 | 1.0000 |  | 0.0297 | 0.3077 |  | 1.0000 |  | TRUE | 0.0000 |
|  |  | MR_Egger | -0.3035(-1.1208, 0.5139) | 0.4682 |  | 66.8735 | 1.0000 |  |  |  |  |  |  |  |  |
|  |  | Weighted_median | -0.0490(-0.6510, 0.5530) | 0.8733 |  |  |  |  |  |  |  |  |  |  |  |
| UPDRS3 | 49 | IVW | -0.0796(-0.1834, 0.0243) | 0.1331 |  | 7.9237 | 1.0000 |  | 0.0100 | 0.2797 |  | 1.0000 |  | FALSE | 0.0013 |
|  |  | MR_Egger | -0.1804(-0.3887, 0.0280) | 0.0964 |  | 6.7275 | 1.0000 |  |  |  |  |  |  |  |  |
|  |  | Weighted_median | -0.1236(-0.2707, 0.0235) | 0.0995 |  |  |  |  |  |  |  |  |  |  |  |
| MMSE | 46 | IVW | -0.0194(-0.2116, 0.1728) | 0.8431 |  | 4.1319 | 1.0000 |  | 0.0040 | 0.8081 |  | 1.0000 |  | FALSE | 0.0000 |
|  |  | MR_Egger | -0.0602(-0.4397, 0.3193) | 0.7573 |  | 4.0722 | 1.0000 |  |  |  |  |  |  |  |  |
|  |  | Weighted_median | 0.0016(-0.2991, 0.3022) | 0.9919 |  |  |  |  |  |  |  |  |  |  |  |
| MOCA | 45 | IVW | -0.0532(-0.6078, 0.5014) | 0.8508 |  | 5.8716 | 1.0000 |  | 0.0415 | 0.3616 |  | 1.0000 |  | FALSE | 0.0002 |
|  |  | MR_Egger | -0.4941(-1.5829, 0.5948) | 0.3787 |  | 5.0212 | 1.0000 |  |  |  |  |  |  |  |  |
|  |  | Weighted_median | -0.3565(-1.1562, 0.4432) | 0.3822 |  |  |  |  |  |  |  |  |  |  |  |
| **Two-hour glucose (2hGlu)** | | | | | | | | | | | | | | | |
| PD risk | 26 | IVW | 1.0273(0.8949, 1.1792) | 0.7024 |  | 16.6698 | 0.8934 |  | 0.0207 | 0.1657 |  | 0.9043 |  | TRUE | 0.0000 |
|  |  | MR_Egger | 0.7784(0.5193, 1.1666) | 0.2367 |  | 14.6262 | 0.9312 |  |  |  |  |  |  |  |  |
|  |  | Weighted_median | 0.9934(0.8317, 1.1865) | 0.9416 |  |  |  |  |  |  |  |  |  |  |  |
| PD AAO | 24 | IVW | -0.0661(-1.0254, 0.8932) | 0.8926 |  | 20.4366 | 0.6155 |  | -0.2121 | 0.0521 |  | 0.6244 |  | TRUE | 0.0000 |
|  |  | MR_Egger | 2.8961(-0.0892, 5.8813) | 0.0704 |  | 16.2187 | 0.8049 |  |  |  |  |  |  |  |  |
|  |  | Weighted_median | -0.2272(-1.5954, 1.1409) | 0.7448 |  |  |  |  |  |  |  |  |  |  |  |
| UPDRS3 | 18 | IVW | -0.0595(-0.2732, 0.1542) | 0.5851 |  | 13.0562 | 0.7324 |  | -0.0291 | 0.2828 |  | 0.7080 |  | TRUE | 0.0000 |
|  |  | MR_Egger | 0.2973(-0.3672, 0.9617) | 0.3935 |  | 11.8208 | 0.7562 |  |  |  |  |  |  |  |  |
|  |  | Weighted_median | -0.0425(-0.3407, 0.2556) | 0.7797 |  |  |  |  |  |  |  |  |  |  |  |
| MMSE | 19 | IVW | 0.0363(-0.3443, 0.4170) | 0.8516 |  | 15.2306 | 0.6461 |  | 0.0445 | 0.3519 |  | 0.6777 |  | TRUE | 0.0000 |
|  |  | MR_Egger | -0.5314(-1.7547, 0.6920) | 0.4064 |  | 14.3145 | 0.6447 |  |  |  |  |  |  |  |  |
|  |  | Weighted_median | 0.0104(-0.4896, 0.5104) | 0.9675 |  |  |  |  |  |  |  |  |  |  |  |
| MOCA | 11 | IVW | 0.6632(-0.5815, 1.9079) | 0.2963 |  | 9.3180 | 0.5022 |  | 0.0251 | 0.8671 |  | 0.4588 |  | TRUE | 0.0000 |
|  |  | MR_Egger | 0.3692(3.9480, 1.4465) | 0.8443 |  | 9.2874 | 0.4112 |  |  |  |  |  |  |  |  |
|  |  | Weighted_median | 1.2725(3.0002, 3.5699) | 0.1488 |  |  |  |  |  |  |  |  |  |  |  |
| **Fasting glucose(FG)** | | | | | | | | | | | | | | | |
| PD risk | 39 | IVW | 0.9452(0.6357, 1.4054) | 0.7807 |  | 47.1767 | 0.1461 |  | 0.0042 | 0.6179 |  | 0.1538 |  | TRUE | 0.0000 |
|  |  | MR_Egger | 0.8007(0.3743, 1.7128) | 0.5702 |  | 46.8561 | 0.1285 |  |  |  |  |  |  |  |  |
|  |  | Weighted_median | 1.0515(0.6225, 1.7762) | 0.8511 |  |  |  |  |  |  |  |  |  |  |  |
| PD AAO | 38 | IVW | 0.4556(-1.9116, 2.8229) | 0.7060 |  | 23.8750 | 0.9531 |  | 0.0750 | 0.1461 |  | 0.9490 |  | TRUE | 0.0000 |
|  |  | MR_Egger | -2.5898(-7.2531, 2.0736) | 0.2836 |  | 21.6680 | 0.9716 |  |  |  |  |  |  |  |  |
|  |  | Weighted_median | -0.9111(-4.4416, 2.6194) | 0.6130 |  |  |  |  |  |  |  |  |  |  |  |
| UPDRS3 | 23 | IVW | -0.2396(-0.8228, 0.3436) | 0.4207 |  | 7.1226 | 0.9988 |  | 0.0011 | 0.9421 |  | 0.9977 |  | TRUE | 0.0000 |
|  |  | MR_Egger | -0.2750(-1.3848, 0.8348) | 0.6322 |  | 7.1172 | 0.9979 |  |  |  |  |  |  |  |  |
|  |  | Weighted_median | -0.3211(-1.0615, 0.4193) | 0.3953 |  |  |  |  |  |  |  |  |  |  |  |
| MMSE | 22 | IVW | 0.6168(-0.3858, 1.6194) | 0.2279 |  | 6.6554 | 0.9987 |  | -0.0231 | 0.3994 |  | 0.9988 |  | TRUE | 0.0004 |
|  |  | MR_Egger | 1.3591(-0.6055, 3.3238) | 0.1902 |  | 5.9139 | 0.9990 |  |  |  |  |  |  |  |  |
|  |  | Weighted_median | 1.0068(-0.3523, 2.3660) | 0.1465 |  |  |  |  |  |  |  |  |  |  |  |
| MOCA | 18 | IVW | 2.1177(-1.0423, 5.2778) | 0.1890 |  | 6.4885 | 0.9893 |  | 0.0080 | 0.9128 |  | 0.9833 |  | TRUE | 0.0201 |
|  |  | MR_Egger | 1.8412(-3.9674, 7.6499) | 0.5432 |  | 6.4761 | 0.9821 |  |  |  |  |  |  |  |  |
|  |  | Weighted_median | 1.7820(-2.0204, 5.5845) | 0.3583 |  |  |  |  |  |  |  |  |  |  |  |
| **Fasting insulin (FI)** | | | | | | | | | | | | | | | |
| PD risk | 58 | IVW | 0.9691(0.6623, 1.4181) | 0.8717 |  | 49.7664 | 0.7406 |  | 0.0028 | 0.7643 |  | 0.7441 |  | TRUE | 0.0000 |
|  |  | MR_Egger | 0.8272(0.2758, 2.4807) | 0.7362 |  | 49.6756 | 0.7116 |  |  |  |  |  |  |  |  |
|  |  | Weighted_median | 0.9906(0.5648, 1.7376) | 0.9739 |  |  |  |  |  |  |  |  |  |  |  |
| PD AAO | 51 | IVW | -2.2556(-5.0465, 0.5353) | 0.1132 |  | 59.9396 | 0.1585 |  | 0.0212 | 0.7596 |  | 0.1646 |  | TRUE | 0.0000 |
|  |  | MR_Egger | -3.4429(-11.5144, 4.6285) | 0.4072 |  | 59.8241 | 0.1383 |  |  |  |  |  |  |  |  |
|  |  | Weighted_median | -1.0782(-5.1072, 2.9508) | 0.5999 |  |  |  |  |  |  |  |  |  |  |  |
| UPDRS3 | 24 | IVW | 0.1719(-0.7996, 1.1434) | 0.7288 |  | 12.6404 | 0.9594 |  | -0.0176 | 0.5430 |  | 0.9456 |  | TRUE | 0.8772 |
|  |  | MR_Egger | 1.0670(-1.9344, 4.0684) | 0.4932 |  | 12.2587 | 0.9518 |  |  |  |  |  |  |  |  |
|  |  | Weighted_median | 0.6508(-0.7006, 2.0022) | 0.3452 |  |  |  |  |  |  |  |  |  |  |  |
| MMSE | 25 | IVW | -0.1346(-1.5561, 1.2868) | 0.8527 |  | 7.2297 | 0.9996 |  | 0.0212 | 0.6219 |  | 0.9995 |  | TRUE | 0.1490 |
|  |  | MR_Egger | -1.1743(-5.4914, 3.1429) | 0.5991 |  | 6.9798 | 0.9995 |  |  |  |  |  |  |  |  |
|  |  | Weighted_median | 0.2150(-1.5888, 2.0187) | 0.8153 |  |  |  |  |  |  |  |  |  |  |  |
| MOCA | 14 | IVW | 0.0570(-5.2813, 5.3953) | 0.9833 |  | 6.0039 | 0.9460 |  | 0.0080 | 0.9555 |  | 0.9415 |  | TRUE | 0.5704 |
|  |  | MR_Egger | -0.3637(-15.7773, 15.0498) | 0.9639 |  | 6.0006 | 0.9161 |  |  |  |  |  |  |  |  |
|  |  | Weighted_median | -1.4444(-8.2524, 5.3635) | 0.6775 |  |  |  |  |  |  |  |  |  |  |  |
| **Glycated hemoglobin levels (HbA1c)** | | | | | | | | | | | | | | | |
| PD risk | 146 | IVW | 0.9903(0.7796, 1.2579) | 0.9362 |  | 178.5737 | 0.0304 |  | -0.0026 | 0.5035 |  | 0.0254 |  | TRUE | 0.0000 |
|  |  | MR_Egger | 1.0962(0.7484, 1.6056) | 0.6379 |  | 178.0177 | 0.0285 |  |  |  |  |  |  |  |  |
|  |  | Weighted_median | 0.9802(0.6685, 1.4373) | 0.9185 |  |  |  |  |  |  |  |  |  |  |  |
| PD AAO | 136 | IVW | 0.0540(-1.3715, 1.4795) | 0.9408 |  | 133.8217 | 0.5125 |  | 0.0063 | 0.7799 |  | 0.5136 |  | TRUE | 0.0000 |
|  |  | MR_Egger | -0.1994(-2.4748, 2.0760) | 0.8639 |  | 133.7433 | 0.4900 |  |  |  |  |  |  |  |  |
|  |  | Weighted_median | 0.6199(-1.8873, 3.1271) | 0.6280 |  |  |  |  |  |  |  |  |  |  |  |
| UPDRS3 | 88 | IVW | -0.0097(-0.4310, 0.4117) | 0.9642 |  | 45.5282 | 0.9999 |  | 0.0012 | 0.8840 |  | 1.0000 |  | TRUE | 0.0000 |
|  |  | MR_Egger | -0.0560(-0.8061, 0.6941) | 0.8841 |  | 45.5068 | 0.9999 |  |  |  |  |  |  |  |  |
|  |  | Weighted_median | 0.1013(-0.5390, 0.7416) | 0.7565 |  |  |  |  |  |  |  |  |  |  |  |
| MMSE | 83 | IVW | 0.4945(-0.1382, 1.1273) | 0.1256 |  | 35.7163 | 1.0000 |  | -0.0144 | 0.2229 |  | 1.0000 |  | TRUE | 0.0000 |
|  |  | MR_Egger | 0.9657(-0.0170, 1.9483) | 0.0576 |  | 34.2075 | 1.0000 |  |  |  |  |  |  |  |  |
|  |  | Weighted_median | 0.6975(-0.2308, 1.6258) | 0.1408 |  |  |  |  |  |  |  |  |  |  |  |
| MOCA | 46 | IVW | -1.1879(-3.3353, 0.9595) | 0.2783 |  | 28.6568 | 0.9725 |  | 0.0266 | 0.4786 |  | 0.9775 |  | TRUE | 0.0000 |
|  |  | MR_Egger | -2.0977(-5.3895, 1.1942) | 0.2183 |  | 28.1460 | 0.9697 |  |  |  |  |  |  |  |  |
|  |  | Weighted_median | -1.2306(-4.3918, 1.9305) | 0.4454 |  |  |  |  |  |  |  |  |  |  |  |
| **Insulin fold change during an oral glucose tolerance test (IFC)** | | | | | | | | | | | | | | | |
| PD risk | 7 | IVW | 1.1312(0.8591, 1.4896) | 0.3797 |  | 3.5551 | 0.7366 |  | 0.0165 | 0.5524 |  | 0.7814 |  | TRUE | 0.0000 |
|  |  | MR_Egger | 0.8550(0.3459, 2.1133) | 0.7482 |  | 3.1500 | 0.6769 |  |  |  |  |  |  |  |  |
|  |  | Weighted_median | 1.0928(0.7700, 1.5511) | 0.6193 |  |  |  |  |  |  |  |  |  |  |  |
| PD AAO | 7 | IVW | 0.1233(-0.1519, 0.3985) | 0.3797 |  | 3.5551 | 0.7366 |  | 0.0165 | 0.5524 |  | 0.8071 |  | TRUE | 0.0000 |
|  |  | MR_Egger | -0.1566(-1.0616, 0.7483) | 0.7482 |  | 3.1500 | 0.6769 |  |  |  |  |  |  |  |  |
|  |  | Weighted_median | 0.0888(-0.2614, 0.4389) | 0.6193 |  |  |  |  |  |  |  |  |  |  |  |
| UPDRS3 | 5 | IVW | -0.2541(-0.7010, 0.1928) | 0.2651 |  | 3.9809 | 0.4086 |  | -0.0528 | 0.2614 |  | 0.4721 |  | TRUE | 0.1671 |
|  |  | MR_Egger | 0.6448(-0.7077, 1.9973) | 0.4190 |  | 2.0761 | 0.5568 |  |  |  |  |  |  |  |  |
|  |  | Weighted_median | -0.4223(-0.9834, 0.1389) | 0.1403 |  |  |  |  |  |  |  |  |  |  |  |
| MMSE | 7 | IVW | 0.0886(-0.6178, 0.7951) | 0.8057 |  | 4.5350 | 0.6047 |  | 0.1272 | 0.1286 |  | 0.6560 |  | TRUE | 0.0048 |
|  |  | MR_Egger | -2.1225(-4.6078, 0.3629) | 0.1550 |  | 1.2271 | 0.9423 |  |  |  |  |  |  |  |  |
|  |  | Weighted_median | 0.0125(-0.8969, 0.9220) | 0.9785 |  |  |  |  |  |  |  |  |  |  |  |
| MOCA | 5 | IVW | 2.1641(-0.5777, 4.9058) | 0.1219 |  | 4.2101 | 0.3783 |  | 0.0638 | 0.8061 |  | 0.4562 |  | TRUE | 0.1307 |
|  |  | MR_Egger | 0.9478(-8.4830, 10.3785) | 0.8564 |  | 4.1117 | 0.2497 |  |  |  |  |  |  |  |  |
|  |  | Weighted_median | 2.0376(-1.5349, 5.6102) | 0.2636 |  |  |  |  |  |  |  |  |  |  |  |
| **Modified Stumvoll Insulin Sensitivity Index (ISI)** | | | | | | | | | | | | | | | |
| PD risk | 10 | IVW | 1.1384(0.8378, 1.5469) | 0.4074 |  | 1.9593 | 0.9921 |  | -0.0025 | 0.9179 |  | 0.9957 |  | TRUE | 0.0000 |
|  |  | MR_Egger | 1.1905(0.4938, 2.8703) | 0.7079 |  | 1.9480 | 0.9826 |  |  |  |  |  |  |  |  |
|  |  | Weighted_median | 1.0821(0.7439, 1.5740) | 0.6800 |  |  |  |  |  |  |  |  |  |  |  |
| PD AAO | 9 | IVW | 0.2405(-1.6529, 2.1338) | 0.8034 |  | 6.6958 | 0.5698 |  | 0.1996 | 0.2582 |  | 0.5114 |  | TRUE | 0.0000 |
|  |  | MR_Egger | -3.1203(-8.7977, 2.5571) | 0.3171 |  | 5.1812 | 0.6379 |  |  |  |  |  |  |  |  |
|  |  | Weighted_median | 0.7484(-1.9227, 3.4194) | 0.5829 |  |  |  |  |  |  |  |  |  |  |  |
| UPDRS3 | 7 | IVW | 0.0918(-0.4547, 0.6383) | 0.7419 |  | 6.7188 | 0.3476 |  | 0.0847 | 0.1470 |  | 0.3906 |  | TRUE | 0.0878 |
|  |  | MR_Egger | -1.1594(-2.6798, 0.3610) | 0.1952 |  | 3.7776 | 0.5819 |  |  |  |  |  |  |  |  |
|  |  | Weighted_median | 0.2164(-0.4319, 0.8648) | 0.5129 |  |  |  |  |  |  |  |  |  |  |  |
| MMSE | 7 | IVW | 0.7216(-0.1969, 1.6402) | 0.1236 |  | 4.7332 | 0.5785 |  | 0.0074 | 0.9247 |  | 0.5411 |  | TRUE | 0.0243 |
|  |  | MR_Egger | 0.5972(-2.0236, 3.2181) | 0.6738 |  | 4.7233 | 0.4506 |  |  |  |  |  |  |  |  |
|  |  | Weighted_median | 0.5761(-0.6346, 1.7867) | 0.3510 |  |  |  |  |  |  |  |  |  |  |  |
| MOCA | 3 | IVW | 0.0518(-3.2917, 3.3954) | 0.9758 |  | 0.9643 | 0.6174 |  | 0.2093 | 0.6035 |  | NA |  | TRUE | 0.1167 |
|  |  | MR_Egger | -2.9374(-11.7548, 5.8799) | 0.6317 |  | 0.4486 | 0.5030 |  |  |  |  |  |  |  |  |
|  |  | Weighted_median | 0.4184(-3.6307, 4.4676) | 0.8395 |  |  |  |  |  |  |  |  |  |  |  |
| **Proinsulin (PROI)** | | | | | | | | | | | | | | | |
| PD risk | 78 | IVW | 0.9763(0.9267, 1.0285) | 0.3661 |  | 76.7050 | 0.4880 |  | -0.0029 | 0.5832 |  | 0.3110 |  | TRUE | 0.0000 |
|  |  | MR_Egger | 0.9955(0.9126, 1.0859) | 0.9195 |  | 76.3997 | 0.4656 |  |  |  |  |  |  |  |  |
|  |  | Weighted_median | 0.9583(0.8843, 1.0385) | 0.2992 |  |  |  |  |  |  |  |  |  |  |  |
| PD AAO | 74 | IVW | 0.0701(-0.3089, 0.4490) | 0.7170 |  | 58.1742 | 0.8971 |  | 0.0078 | 0.8324 |  | 0.8994 |  | TRUE | 0.0000 |
|  |  | MR_Egger | 0.0127(-0.6384, 0.6638) | 0.9696 |  | 58.1291 | 0.8816 |  |  |  |  |  |  |  |  |
|  |  | Weighted_median | 0.1440(-0.4318, 0.7199) | 0.6239 |  |  |  |  |  |  |  |  |  |  |  |
| UPDRS3 | 66 | IVW | 0.0266(-0.0642, 0.1174) | 0.5662 |  | 50.8169 | 0.9012 |  | -0.0084 | 0.4240 |  | 0.8994 |  | TRUE | 0.0000 |
|  |  | MR_Egger | 0.0834(-0.0822, 0.2491) | 0.3271 |  | 50.1694 | 0.8968 |  |  |  |  |  |  |  |  |
|  |  | Weighted_median | 0.0162(-0.1208, 0.1533) | 0.8162 |  |  |  |  |  |  |  |  |  |  |  |
| MMSE | 67 | IVW | 0.0512(-0.1068, 0.2092) | 0.5254 |  | 66.5332 | 0.4585 |  | 0.0220 | 0.1980 |  | 0.4660 |  | TRUE | 0.0000 |
|  |  | MR_Egger | -0.1048(-0.3877, 0.1781) | 0.4704 |  | 64.8417 | 0.4822 |  |  |  |  |  |  |  |  |
|  |  | Weighted_median | -0.0864(-0.3138, 0.1410) | 0.4566 |  |  |  |  |  |  |  |  |  |  |  |
| MOCA | 31 | IVW | 0.0537(-0.4759, 0.5834) | 0.8424 |  | 24.8797 | 0.7309 |  | 0.0777 | 0.2387 |  | 0.7469 |  | TRUE | 0.0000 |
|  |  | MR_Egger | -0.4679(-1.4694, 0.5335) | 0.3673 |  | 23.4327 | 0.7564 |  |  |  |  |  |  |  |  |
|  |  | Weighted_median | -0.1429(-0.8934, 0.6075) | 0.7089 |  |  |  |  |  |  |  |  |  |  |  |

N SNPs: number of single nucleotide polymorphisms in the instrument. IVW: Inverse variance weighted. MR: Mendelian randomization. MR-PRESSO: Mendelian Randomization Pleiotropy RESidual Sum and Outlier. OR: Odds ratio. CI: confidence interval. Beta: MR effect estimate. se: standard error of MR effect estimate. P: P-value. PD: Parkinson's disease. UPDRS3: Unified Parkinson's Disease Rating Scale part III. MMSE: Mini-Mental State Examination. MoCA: Montreal Cognitive Assessment. Describing PD risk results using OR (95% CI) and UPDRS3/MMSE/MOCA results using Bete (95% CI).

**Table S4** MR assessment for the significant results of reverse MR analyses.

| **Outcome** | **N  SNVs** | **MR  analysis** | | |  | **Heterogeneity  Test** | |  | **MR-Egger pleiotropy  Test** | |  | **MR PRESSO Test** |  | **Directionality  Test** | |
| --- | --- | --- | --- | --- | --- | --- | --- | --- | --- | --- | --- | --- | --- | --- | --- |
|  |  | **Method** | **Estimate (95% CI)** | ***P*** |  | **Q value** | ***P*** |  | **Egger intercept** | ***P*** |  | **Global Test *P*** |  | **Correct directionaliy** | ***P*** |
| TIDM | 10 | IVW | 0.9347(0.8657, 1.0092) | 0.0844 |  | 3.9055 | 0.9175 |  | 0.0097 | 0.4600 |  | 0.0000 |  | TRUE | 0.0000 |
|  |  | MR_Egger | 0.8775(0.7353, 1.0473) | 0.1856 |  | 3.3031 | 0.9139 |  |  |  |  |  |  |  |  |
|  |  | Weighted_median | 0.9155(0.8297, 1.0101) | 0.0786 |  |  |  |  |  |  |  |  |  |  |  |

N SNPs: number of single nucleotide polymorphisms in the instrument. IVW: Inverse variance weighted. MR: Mendelian randomization. MR-PRESSO: Mendelian Randomization Pleiotropy RESidual Sum and Outlier. OR: Odds ratio. CI: confidence interval. P: P-value. PD: Parkinson's disease.

**Table S5** Multivariable MR results after adjusting for the Anti-diabetic drugs.

| **Medications** | **Outcome** | **Adjustments** | **N SNP** | **Methods** | **Causal effect** | |  | **Heterogeneity** | |  | **Pleiotropy** | |
| --- | --- | --- | --- | --- | --- | --- | --- | --- | --- | --- | --- | --- |
|  |  |  |  |  | **OR (95%CI)** | **p** |  | **Q value** | **p** |  | **Intercept** | **p** |
| Type 1 diabetes  (T1DM) | PD risk | Drug used in diabetes | 134 | IVW | 0.9812(0.9324, 1.0325) | 0.4740 |  | 61.8836 | 0.2742 |  | 61.5000 | 0.2547 |
|  |  |  |  | MR_Egger | 0.9831(0.9216, 1.0488) | 0.6130 |  | 61.8723 | 0.2442 |  |  |  |
|  |  |  |  | Weighted_median | 0.9950(0.9272, 1.0678) | 0.8900 |  |  |  |  |  |  |
|  |  | Diabetes, insuline treatment | 126 | IVW | 0.9822(0.9463, 1.0194) | 0.3380 |  | 63.9780 | 0.8900 |  | 63.92387 | 0.8747 |
|  |  |  |  | MR_Egger | 0.9970(0.9456, 1.0512) | 0.9030 |  | 63.3573 | 0.8851 |  |  |  |
|  |  |  |  | Weighted_median | 0.9881(0.9371, 1.0418) | 0.6590 |  |  |  |  |  |  |
|  |  | Metformin | 126 | IVW | 1.0000(0.9825, 1.0178) | 0.9930 |  | 117.5260 | 0.1395 |  | 117.5186 | 0.1249 |
|  |  |  |  | MR_Egger | 1.0010(0.9777, 1.0248) | 0.9240 |  | 117.4999 | 0.1252 |  |  |  |
|  |  |  |  | Weighted_median | 0.9881(0.9594, 1.0176) | 0.4300 |  |  |  |  |  |  |

N SNPs: number of single nucleotide polymorphisms in the instrument. IVW: Inverse variance weighted. MR: Mendelian randomization. MR-PRESSO: Mendelian Randomization Pleiotropy RESidual Sum and Outlier. OR: Odds ratio. CI: confidence interval. P: P-value. PD: Parkinson's disease.
